# Cellulose Nanocrystal Templated Graphene Nanoscrolls for High Performance Supercapacitors and Hydrogen Storage: An Experimental and Molecular Simulation Study

**DOI:** 10.1038/s41598-018-22123-0

**Published:** 2018-03-01

**Authors:** Prodyut Dhar, Surendra Singh Gaur, Amit Kumar, Vimal Katiyar

**Affiliations:** Department of Chemical Engineering, Indian Institute of Technology Guwahati, Guwahati, 781039 Assam India

## Abstract

Graphene nanoscrolls (GNS), due to their remarkably interesting properties, have attracted significant interest with applications in various engineering sectors. However, uncontrolled morphologies, poor yield and low quality GNS produced through traditional routes are major challenges associated. We demonstrate sustainable approach of utilizing bio-derived cellulose nanocrystals (CNCs) as template for fabrication of GNS with tunable morphological dimensions ranging from micron-to-nanoscale(controlled length < 1 μm or >1 μm), alongwith encapsulation of catalytically active metallic-species in scroll interlayers. The surface-modified magnetic CNCs acts as structural-directing agents which provides enough momentum to initiate self-scrolling phenomenon of graphene through van der Waals forces and π-π interactions, mechanism of which is demonstrated through experimental and molecular simulation studies. The proposed approach of GNS fabrication provides flexibility to tune physico-chemical properties of GNS by simply varying interlayer spacing, scrolling density and fraction of encapsulated metallic nanoparticles. The hybrid GNS with confined palladium or platinum nanoparticles (at lower loading ~1 wt.%) shows enhanced hydrogen storage capacity (~0.2 wt.% at~20 bar and ~273 K) and excellent supercapacitance behavior (~223–357 F/g) for prolonged cycles (retention ~93.5–96.4% at ~10000 cycles). The current strategy of utilizing bio-based templates can be further extended to incorporate complex architectures or nanomaterials in GNS core or inter-layers, which will potentially broaden its applications in fabrication of high-performance devices.

## Introduction

Graphene nanoscrolls (GNS) are one of the most emerging carbon-based nanomaterials which have been the focus of several recent studies due to their interesting physico-chemical properties and one-dimensional topological structures produced through rolling of two-dimensional graphene sheets. GNS are morphologically similar to multiwalled carbon nanotubes(CNTs); however, their open end structures, variable scrolling density and tunable inter-layer spacing provides a unique opportunity to inherit favorable properties of both CNTs and graphene^1,2^. The tubular nanochannels and high porosity present in GNS provides pathway for intercalation of ionic species in its inter-galleries resulting in higher electrical conductivities compared to graphene with practical applications in electrochemical charge storage devices^3^ and electronics^4^. In literature, molecular simulation-based studies on the formation of GNS and theoretical predictions of its properties, have been extensively studied^5,6^. However, self-assembly of GNS through real-time experiments is technically difficult to achieve. Despite the unique properties of GNS, it has been shown to undergo unwrapping leading to formation of defects^7^, due to which studies on potential applications of GNS in hydrogen (H_2_) storage devices^8^, CO_2_ capture^9^ and catalysis have been only possible through simulation studies. Some of the investigations for fabrication of GNS are based on self-assembly of graphene through controlled sonication approaches, Langmuir-Blodgett^10^, cyclic freezing-thawing^11,12^, chemical vapor deposition^13^ and ordered exfoliation or intercalation of graphitic inter-layers^14^. The aforementioned techniques are usually energy intensive processes utilizing harsh reaction conditions or chemicals with lower yields/poor quality of GNS and have issues related to environmental sustainability, which needs to be addressed through strategic modifications of GNS fabrication routes.

The major challenges in GNS processing is to effectively control the morphological dimensions^15^ and produce it with higher monodispersity index, yield and quality through scalable approaches. From the application point of view, GNS with higher aspect ratio are preferred in case of energy storage devices (such as capacitors or batteries), due to their superior electrical transport behaviour over longer length scales and ability to form electric double layer capacitors^3^. Moreover, GNS with low aspect ratio and higher degree of porosity are preferred for potential applications in catalysis and hydrogen (H_2_) storage devices^8^ due to their ability to entrap H_2_ molecules in nanochannels. Introduction of catalytically active nanomaterials in core of GNS is expected to improve the electrochemical storage through formation of pseudocapacaitance^16^ and enhanced H_2_ uptake through spillover mechanism^17^. However, production of GNS with distinct morphological dimensions have been seldom reported through simulation studies^18^ but experimental techniques to synthesize size tunable GNS (from nano to micron-scale dimensions) with various metallic moieties encapsulated in its core as well as inter-galleries, is a challenging tasks and needs to be investigated.

In this paper, a facile and sustainable approach for fabrication of GNS with tunable dimensions and catalytically active metallic intercalates has been developed, through utilization of CNCs derived from bamboo stems as templates. CNCs are crystalline domains of cellulose with rod-like morphology which contains abundant hydroxyl groups, high surface area, tunable aspect ratio, higher modulus, surface reactivity, and interesting optical properties due to its non-toxic behavior, improved bio-compatibility and biodegradability, which finds growing interest in both fundamental as well as applied research. Surface modified magneto-responsive CNCs shows presence of both external stimuli forces (magnetic or electrostatic interactions) and acts as structural directing agents which is expected to have synergistic effect, leading to improved scrolling efficiency of graphene sheets with reduced defects. The variation in inherent characteristics of CNCs (initial precursor) in terms of structural, morphological behaviour and functionality (modified with magnetic or catalytically active metallic species), provides us a unique opportunity to tune physico-chemical properties of GNS. The rolling of graphene sheets into GNS in presence of magneto-responsive CNCs is a complex phenomenon, mechanism of which has been understood through combined experimental as well as molecular simulation studies. Furthermore, as a proof of concept, two different catalytically active platinum (Pt) and palladium (Pd) nanoparticles are confined in the core of GNS and their effect on H_2_ storage and electrochemical performance as supercapacitors has been studied. Therefore, this study utilizes CNCs as a powerful tool to induce self-assembly of graphene to manipulate the structural and chemical properties of GNS through selective confinement of metallic nanoparticles, which may provide it exceptional features for potential high performance applications.

## Results

### Fabrication of graphene nanoscrolls (GNS) using Fe_2_O_3_-CNC as templates: Effect of CNC aspect ratio

**Figure 1:**
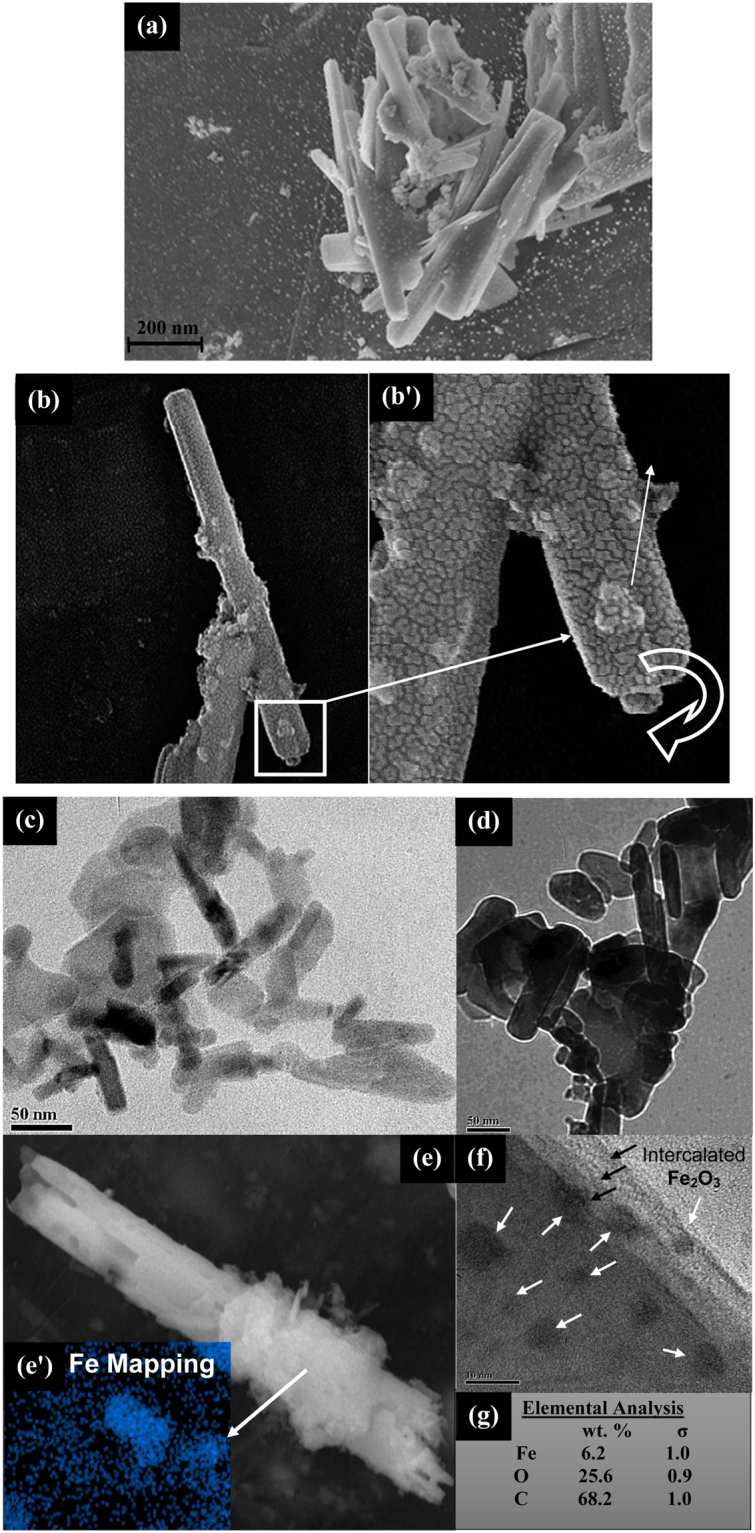
(**a**) FESEM images of the Fe_2_O_3_-NS fabricated using low aspect ratio Fe_2_O_3_-CNCs as initial precursor, (**b**) and (**b’**) High resolution FESEM micrographs of a Fe_2_O_3_-NS (at magnification ~230KX) shows the presence of the Fe_2_O_3_ nanoparticles in its core and scrolling of the graphene sheets to form an open end GNS, (**c**) and (**d**) TEM micrographs of the low aspect ratio Fe_2_O_3_-NS, (**e**) and (**e’**) shows the EDX map of the selected region in the FESEM micrographs for a Fe_2_O_3_-NS encapsulated with Fe_2_O_3_ nanoparticles (**f**) TEM image of an edge of Fe_2_O_3_-NS which shows number of graphene layers (marked with black arrows) and the encapsulated Fe_2_O_3_ nanoparticles (marked with white arrows) and (**g**) compositional analysis of the Fe_2_O_3_-NS.

The surface modified magneto-responsive CNCs acts as a template for scrolling of graphene sheets to form GNS with tunable morphology and inter-layers, which depends upon the aspect ratio of initial substrate. In this study, after exfoliation, the graphene sheets were sonicated for a fixed duration of time (~30 mins in methanol) which leads to the formation of size-specific GRO sheets with dimensional area of 0.56 ± 0.3 µm^2^ (as shown in Fig. S1(a and b)). The FESEM micrographs and collected TEM micrographs shows the presence of several bunches of the GRO sheets, which further confirms its similar morphological dimensions. The fabricated Fe_2_O_3_-CNCs (as discussed in Supporting Information, Part I) have high fractions (~35 wt%) of maghemite nanoparticles decorated on its surface, which integrates magnetic moment of ~43 emu/g alongwith the distinct rod-like morphology (length ~745 ± 63 nm and diameter ~56 ± 8 nm), makes it an ideal candidate for GNS fabrication (Fig. S2(a–g)). It is noteworthy to state that due to polydisperse nature of CNCs and graphene sheets, it is difficult to control the dimensions of GNS, however, the main objective of this study is to fabricate GNS with length in range of micron and nano scales distinctively (with controlled length < 1 μm or >1 μm), from the application point of view (as discussed in Introduction). FESEM and TEM micrographs shows bunch of GNS (Fig. 1(a,b and c)) fabricated using low aspect ratio Fe_2_O_3_-CNCs as templates (Fe_2_O_3_-NS-LR), with length ~750 ± 230 nm and diameter ~174 ± 50 nm. Interestingly, it was observed that dimensions of fabricated Fe_2_O_3_-NS especially its length is found to be almost similar to that of Fe_2_O_3_-CNCs (which is further confirmed from the collected TEM micrographs, Fig. S4(a)). However, the increased diameter is probably due to adherence of multiple epoxidized Fe_2_O_3_-CNCs onto graphene sheets during the GNS formation process. High resolution FESEM micrographs (Fig. 1(b and b’)) of individual Fe_2_O_3_-NS shows the presence of Fe_2_O_3_ nanoparticles confined in its core with end tip showing the scrolling of graphene sheets with open ends. The investigations of HR-TEM micrographs of Fe_2_O_3_-NS edge shows the presence of multiple-layers of graphene sheets (marked with black arrows) with intercalated Fe_2_O_3_ nanoparticles in its core as well as inter-layers (marked with white arrows, Fig. 1(f)). Further, EDX mapped image of selected Fe_2_O_3_-NS shows the presence of Fe_2_O_3_ nanoparticles with a composition of ~6.2 wt.% Fe (Fig. 1(e and g)). Therefore, from morphological and elemental analysis it could be confirmed that Fe_2_O_3_-CNCs acts as structural directing agent which provides enough momentum to initiate scrolling of graphene sheets to form Fe_2_O_3_-NS with encapsulated Fe_2_O_3_ nanoparticles.

**Figure 2:**
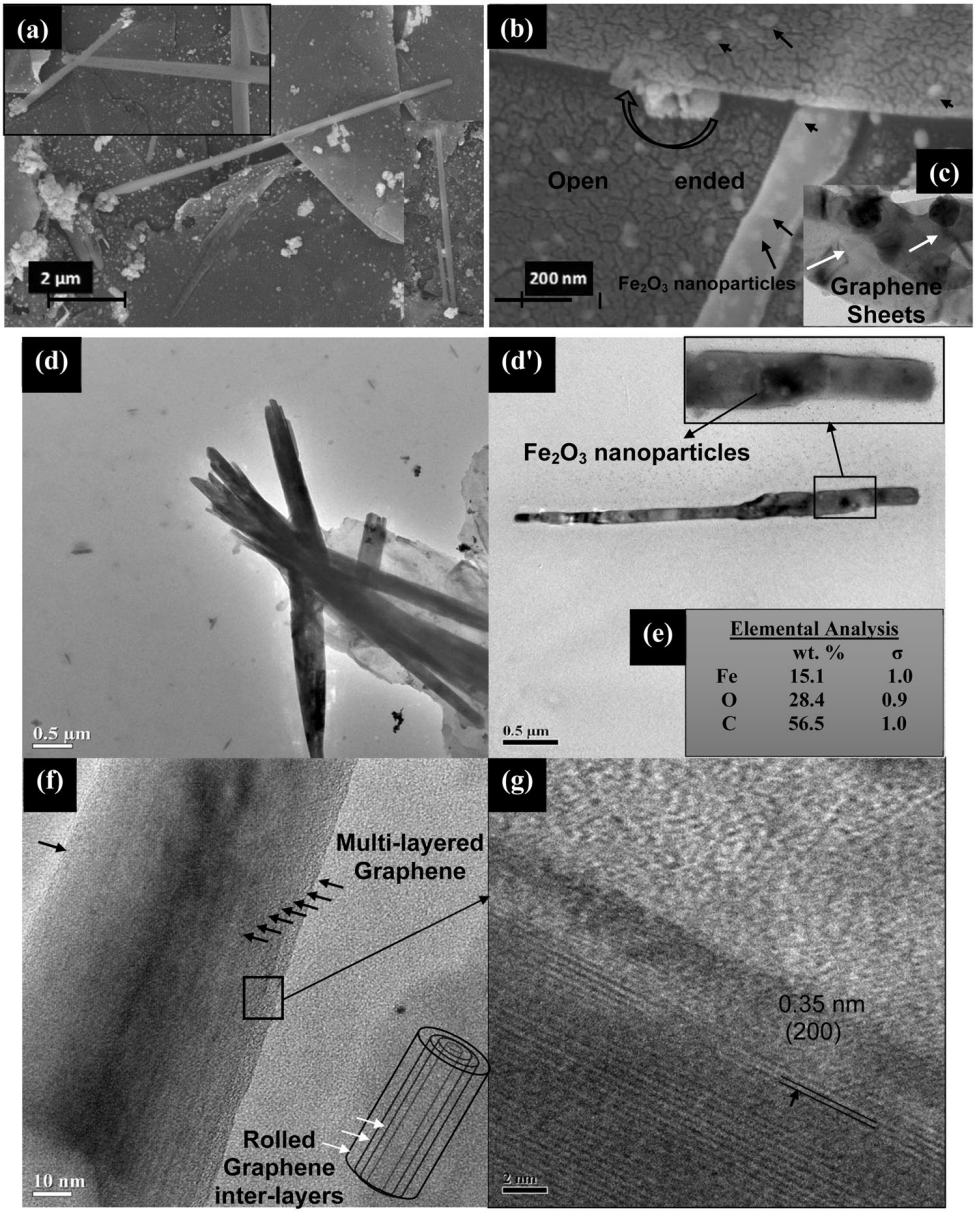
(**a**) FESEM micrographs of Fe_2_O_3_-NS fabricated using high aspect ratio Fe_2_O_3_-CNCs as the structural directing templates, (**b**) High resolution FESEM micrographs (at a resolution of ~210KX) shows the encapsulated Fe_2_O_3_ nanoparticles and the scrolling of the graphene sheets with an open end, (**c**) TEM micrographs shows the wrapping of the Fe_2_O_3_ nanoparticles with a single layer of graphene sheets, (**d)** and (**d’**) TEM micrographs of a bunch of Fe_2_O_3_-NS fabricated using high aspect ratio Fe_2_O_3_-CNCs as template, (**e**) elemental composition of the Fe_2_O_3_-NS, (**f**) Fe_2_O_3_-NS with the presence of number of layers of graphene sheets rolled on it (as marked with the black arrows) and (**g**) HRTEM image of the selected region with the presence of the lattice fringes of 0.35 nm corresponding to (200) plane of graphene.

To further investigate the effect of CNC dimensions on GNS formation, high aspect ratio precursor Fe_2_O_3_-CNC-HR is formed using the CNCs derived from filter paper^19^ (as detailed in Supporting Information, Part 1). Utilization of Fe_2_O_3_-CNC-HR (with length ~2.8 ± 0.8 μm and diameter ~82 ± 18 nm) (Fig. S3) as template, lead to the formation of Fe_2_O_3_-NS with length ~4.5 ± 1.2 μm and diameter ~0.37 ± 0.21 μm as evident from FESEM and TEM micrographs (Fig. 2(a and d)), respectively. It could be observed that the fabricated Fe_2_O_3_-NS-HR are in micron dimensions (with length > 1 μm) which have successfully inherited the structural properties of Fe_2_O_3_-CNC-HR (as also confirmed through the collected FESEM micrographs shown in Fig. S4(b)). Further, Fe_2_O_3_-NS shows the presence of the open ends suggesting its hollow nature (Fig. 2(b)) which have thin layer of graphene sheets wrapped on Fe_2_O_3_ nanoparticles (Fig. 2(c and d’)). The compositional analysis of Fe_2_O_3_-NS-HR contains ~15 wt% of Fe_2_O_3_ nanoparticles (Fig. 2(e)) which is higher in comparison to Fe_2_O_3_-NS-LR due to adsorption of higher fragments of Fe_2_O_3_-CNCs onto graphene sheets during GNS formation. Similarly, HRTEM micrographs in case of Fe_2_O_3_-NS-HR (Fig. 2(f)) shows presence of higher number of multi-layered graphene sheets rolled on it compared to Fe_2_O_3_-NS-LR. This is probably due to high fractions of Fe_2_O_3_ nanoparticles in the inner cores of Fe_2_O_3_-NS-HR which results in higher magnetic moment, providing enough torque on graphene sheets to overcome the threshold energy barrier and undergo scrolling phenomenon to form multi-layered structure onto Fe_2_O_3_-CNCs. Similar theoretical observations was also reported by Wang *et al*. (2015)^13^, on utilization of low aspect ratio carbon nanotubes as structural directing agent which failed to initiate the nanoscroll formation process, due to its lowered van der Waals interaction energy. Figure 2(g), shows the lattice fringes with interlayer spacing of ~0.35 nm represents (200) crystal planes of graphene sheets, confirming its presence. Therefore, it could be inferred that both morphology and density of scrolled graphene in Fe_2_O_3_-NS can be remotely controlled by simply tuning the density of maghemite nanoparticles as well as magnetic moment/dimensions of Fe_2_O_3_-CNCs (template), which provides a facile approach of fabricating GNS with tunable properties depending upon the targeted applications.

**Figure 3:**
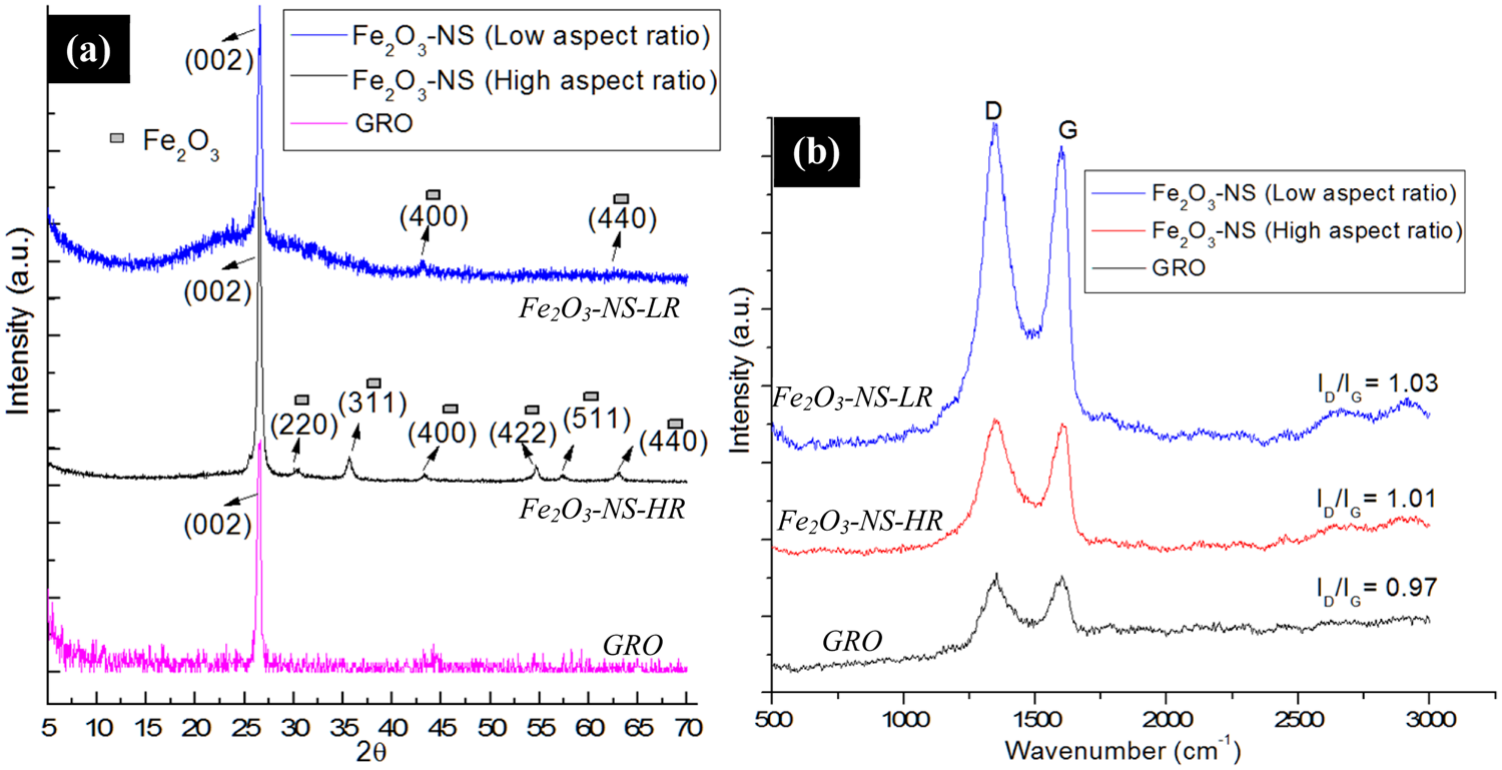
(**a**) XRD diffractograms and (**b**) Raman spectroscopy of the Fe_2_O_3_-NS fabricated using various aspect ratio of the Fe_2_O_3_-CNCs as the structural directing templates.

Figure 3(a), shows XRD diffractograms of Fe_2_O_3_-NS formed with different aspect ratio which shows presence of peak at 2θ = 26.5° corresponding to graphene representing (002) planes^11^. The XRD spectra for Fe_2_O_3_-NS-HR, shows the presence of distinctive peaks at 2θ = 30.4°, 35.6°, 43.2°, 54.5°, 57.3° and 63.1° corresponding to (220), (311), (400), (422),(511) and (440) planes of Fe_2_O_3_ nanoparticles^20^ respectively, which confirms their presence in core of GNS. However, in case of Fe_2_O_3_-NS-LR, presence of small intensity peaks at 2θ = 43.2° and 62.5° represents (400) and (440) planes of Fe_2_O_3_ nanoparticles, respectively. The low intensity XRD peaks of Fe_2_O_3_ in case of Fe_2_O_3_-NS-LR, is probably due to presence of low fractions of Fe_2_O_3_ nanoparticles alongwith presence of graphene layers covering its surface. Therefore, XRD diffractograms of both high and low aspect ratio GNS confirms presence of Fe_2_O_3_ nanoparticles in scrolled graphene sheets. The SAED pattern of Fe_2_O_3_-NS interestingly shows the presence of sharp hexagonal spots which are concentric in nature with the presence of equatorial line showing that the diffraction pattern of GNS consists of intense spots along a line perpendicular to the needle axis. The presence of the multiple and concentric diffraction patterns (marked with dotted white lines) suggests that graphene sheets have undergone scrolling phenomenon to form a multilayered rolled structure, which arises due to the diffractions from the individual set of rolled graphene sheets^21^ present in the Fe_2_O_3_-NS. The presence of the equatorial line along the spots {0004} and {0002}^22^, suggest that the presence of the parallel graphene layers with stacking periodicity (and perpendicular orientation with respect to electron beam) which confirms the formation of Archimedean spiral in the inner core of the Fe_2_O_3_-NS. Figure S5(b), shows the SAED pattern of the Fe_2_O_3_-NS which shows the presence of the well-defined diffraction spots with an ordered continuity along the equatorial line. Information on the degree of packing of the multi-layered GRO sheets in the Fe_2_O_3_-NS can be obtained by considering the equatorial line of the SAED pattern. On the marked equatorial line, four spots can be distinguisly visualized from which the profile of the intensities was calculated to determine the structure and lattice parameter of the Fe_2_O_3_-NS. From the electron diffraction pattern, presence of the concentric hexagons represents the packing of the multi-layered graphene sheets which have occurred during the scrolling of the GRO sheets. Further, in line with the previously reported literature it was observed that the equatorial line rotates along the centre of polygon with the presence of high intensity spots present at its curves (towards radial directions), suggesting, the circular morphology of the Fe_2_O_3_-NS (present at its cross-section). Evaluation of the SAED pattern and the intensity profiles (Fig. S5(b and c)) of the selected section of Fig. S5(a), suggests the presence of two bundles of Fe_2_O_3_-NS with interlayer spacing of 0.318 nm.

The epoxidation of Fe_2_O_3_-CNCs followed by scrolling of graphene sheets to form Fe_2_O_3_-NS, are confirmed from the FTIR and Raman spectroscopy studies. As reported in our earlier studies^23^, the abundant hydroxyl groups in CNCs provides sites for the precipitation of Fe_2_O_3_ nanoparticles on its surface, which was confirmed from the presence of peak at ~620 cm^−1^ and shoulder peak at ~700 cm^−1^ corresponding to Fe-O vibrations, absence of the free hydroxyl peaks of CNC at 3280 cm^−1^ and shift of peak (-OH bending) at ~1646 cm^−1^ (in CNCs) to 1652 cm^−1^ (Fig. S5(a)). Further, on modifications epoxidized Fe_2_O_3_-CNCs shows the presence of new peaks at ~3434 cm^−1^ corresponding to the C-H stretching vibration of epoxy groups in epichlorohydrin and with formation of small peak at ~1290 cm^−1^ (C-O-C stretching) confirms the presence of epoxidized ring in modified Fe_2_O_3_-CNCs^24^. The FTIR spectrograph of the GRO sheets (after sonication in presence of the hydrogen peroxide) shows the presence of peaks at 3550 cm^−1^,1200 cm^−1^, 1070 cm^−1^ and 1720 cm^−1^ corresponding to O-H stretching, C-O epoxy stretching, C-O alkoxy stretching and C=O carbonyl stretching vibrations^25^. In Fe_2_O_3_-CNC-NS, the new peaks at ~3504 cm^−1^ corresponds to -OH stretching and 1595 cm^−1^ denotes skeletal vibrations from graphitic rings of GRO sheets^25^ alongwith the decrease in intensity of peaks at 1062 cm^−1^, 1114 cm^−1^ and 1158 cm^−1^ (representing -C-O-C asymmetric stretching of β-glucosidic linkage, C-OH stretching in plane at C-6, and -C-O-C asymmetric bending of β-glucosidic linkage) confirms scrolling of graphene onto Fe_2_O_3_-CNCs by ring opening in epoxidized Fe_2_O_3_-CNC through formation of C-O-C bonding. On calcination of Fe_2_O_3_-CNC-NS, it was observed that the intensity of the various oxygen functionalities suppressed which is possibly due to the reduction of the partially oxidized GRO sheets to graphene^25^. The Raman spectra of GRO sheets shows a G-band at ~1582 cm^−1^ and D-band at ~1354 cm^−1^ with an I_D_/I_G_ ratio of ~0.97 (Fig. 3(b)). The epoxidation of Fe_2_O_3_-CNCs followed by its reaction with partially oxidized graphene (GRO) sheets brings it in close proximity which is a necessary step to initiate the nanoscrolls formation process that occurs through п-п interactions and magnetic manipulation^26^. Due to such phenomenon, the epoxidized Fe_2_O_3_-CNCs adheres to inner core of GNS through sp^3^-hybridization, which leads to formation of open-ended GNS with more defects that could be evaluated through values of I_D_/I_G_ ratio^11^. However, it was observed that presence of such scrolling phenomenon was completely absent in presence of the unmodified CNCs as shown in Fig. S5(d), with the presence of bunch of CNCs lying on the surface of GRO sheets (Fig. S5(d’)). Therefore, it could be concluded that the Fe_2_O_3_-CNCs are responsible to initiate the scrolling of the graphene sheets resulting in the formation of Fe_2_O_3_-NS. Further, the effect of Fe_2_O_3_-CNCs on morphology of GNS also lead to significant alteration in Raman spectra with increased I_D_/I_G_ ratio ~1.03 for Fe_2_O_3_-NS-LR in comparison to Fe_2_O_3_-NS-HR ~1.01. As evident from morphological studies (Figs 1 and 2(f)), lower I_D_/I_G_ values for Fe_2_O_3_-NS-HR suggests lower defects that will increase graphene layers in GNS compared to Fe_2_O_3_-NS-LR, which have higher defects alongwith low scrolling density due to its increased sp^3^-hybridization. Further form Raman spectroscopy, it could be confirmed that GNS with variable scrolling density and tunable morphological dimensions can be fabricated by simple altering the aspect ratio of CNCs.

### Mechanism for graphene nanoscrolls formation using Fe_2_O_3_-CNCs as templates: Molecular simulation studies

**Figure 4:**
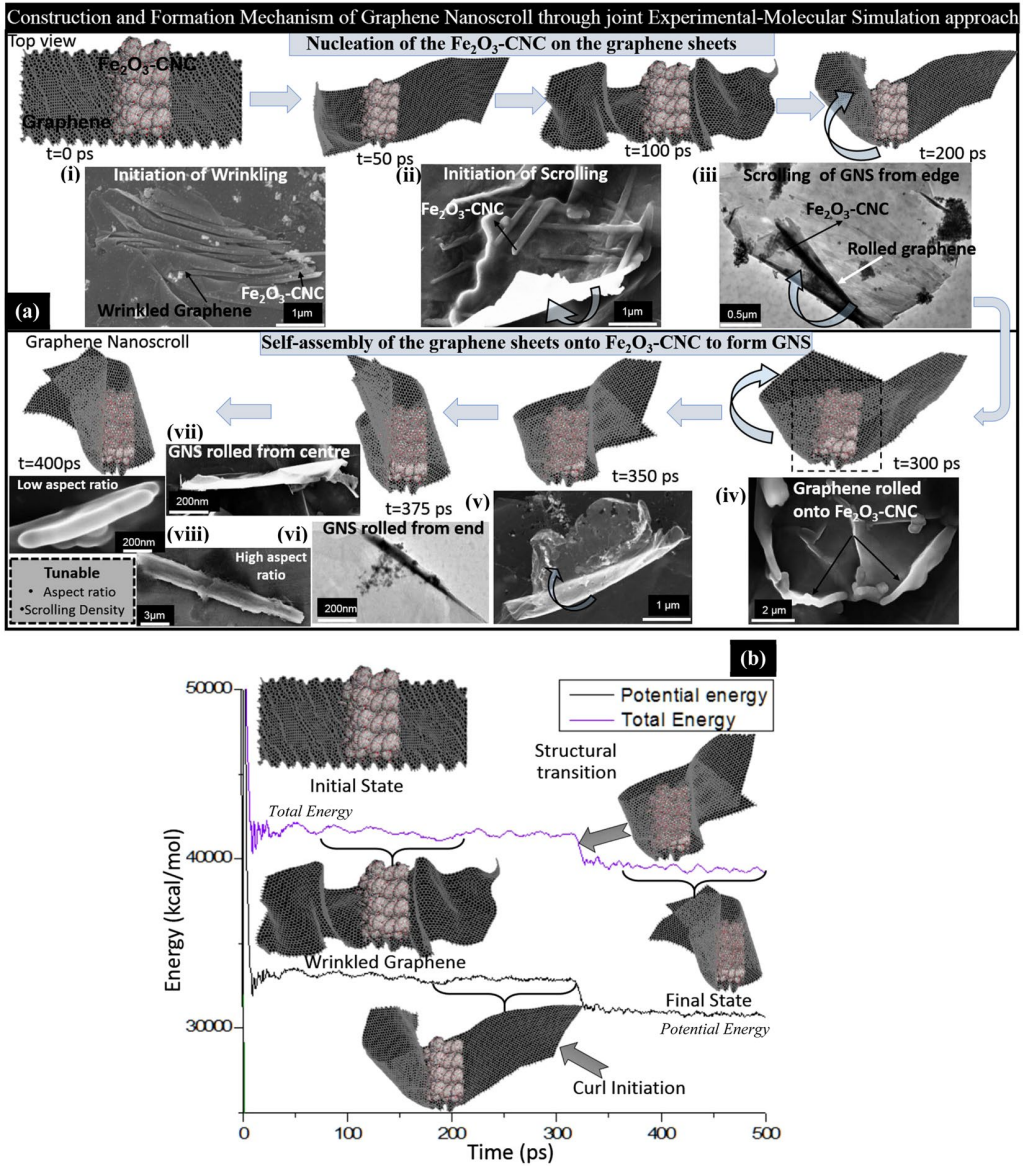
(**a**) Molecular simulation studies showing snapshots of dynamic self-assembly of graphene on Fe_2_O_3_-CNCs along with their evidence through electron micrographs at different time-scales and stages as mentioned in images (i), (ii), (iii), (iv), (v), (vi), (vii) and (viii). (**b**) Evolution of the total and potential energy plots for the formation of Fe_2_O_3_-NS using Fe_2_O_3_-CNCs as templates at different time scales with the insets showing the conformational changes at different times.

In order to understand the mechanism and driving forces responsible for formation of GNS onto Fe_2_O_3_-CNCs substrate, molecular simulation studies are carried out which is jointly corroborated with the experimental findings (electron micrographs obtained during optimization of GNS fabrication process) (Fig. 4(a)). As addressed from experimental studies that the effect of aspect ratio of initial substrate Fe_2_O_3_-CNCs and their high magnetic behaviour are responsible for the formation of nanoscrolls is further verified through MD simulation studies. As a typical representation of experimental studies, a molecular model of Fe_2_O_3_-CNC with a low aspect ratio (~13), similar magnetic properties and loading fractions of Fe_2_O_3_ nanoparticles (~35 wt%) was prepared followed by its geometry optimization to obtain energy minimized morphology. The Fe_2_O_3_-CNC was placed at the centre of a graphene sheet (with length ~116.2 Å and width ~58.1 Å) along the axis of Fe_2_O_3_-CNC (Fig. 4a(i)). On geometry optimization, Fe_2_O_3_-CNC approaches closer to the surface of graphene sheet because of the presence of favourable attractive forces in between the two moieties. Thereafter, MD study was carried out on the prepared structure over a time period of ~400 ps to observe conformational changes as well as understand the effect of van der Waals (vdW) interaction forces between graphene sheets and Fe_2_O_3_-CNC. The formation and growth mechanism of Fe_2_O_3_-NS can be classified into two steps: firstly the nucleation of Fe_2_O_3_-CNCs onto graphene sheets followed by the second step in which self-scrolling of graphene on its surface to form GNS takes place over a period of time to form tight scrolls (see video S1 in Supplementary information). Initially at t = 0 ps, presence of Fe_2_O_3_-CNC results in formation of discontinuous wrinkled structures on the graphene sheets indicating thermodynamic instability until t = 100 ps. It is also evident from the total energy and vdW interaction energy plots which shows a slight drop till t = 20 ps due to improved interactions and thereafter remains almost constant till t = 200 ps. Similar observations on wrinkled state of graphene sheets with bunch of Fe_2_O_3_-CNCs adhered on its surface are evident from experimental studies as shown in Fig. 4(a). After time period of t = 200 ps, the initial curl of graphene sheet starts to occur on the Fe_2_O_3_-CNC surface from one side of edge. The initiation of curl of graphene sheet is visible from TEM micrographs (Fig. 4a(ii)) which shows the formation of twisted morphology along the ends of Fe_2_O_3_-CNCs (marked in blue arrow) adhered to the surface of GRO. In next 300 ps, the graphene sheet starts to roll on the surface of Fe_2_O_3_-CNC from one end (left side first) and tries to adjust to obtain the best possible stable configuration (as shown in Fig. 4a(iii and iv)). It is observed that the graphene layers slide over the Fe_2_O_3_-CNC slowly during which the total and potential energy remain almost constant. The intermediate step for partially scrolled graphene sheet layers onto Fe_2_O_3_-CNC surface can be observed from the FESEM micrographs (marked with blue arrow as shown in Fig. 4a(iv and v)). After t = 300 ps, structural transition of the 2D planar structure of graphene takes place to form a 1D tubular GNS due to strong interaction between the graphene sheets and Fe_2_O_3_-CNC. The transition is also evident from the sharp drop in potential as well as total energy values (as shown in Fig. 4(b)) during the time span of ~320–350 ps, which suggest that the system has undergone significant structural alteration to attain a minimum energy state. In this state, the graphene sheet completely covers the surface of Fe_2_O_3_-CNC and acquires the structural configuration of the rod-like morphology of the Fe_2_O_3_-CNC (Fig. 4a(vi and vii)). Due to presence of high loadings of Fe_2_O_3_ nanoparticles and its improved interfacial interaction with the graphene sheets undergoes scrolling as visualized from the rolled edges to form a complete nanoscroll morphology. From the molecular simulation studies, it was observed that the energy of the intermediate state of bending for the graphene sheet of length of ~116 Å, was found to be 1021 kcal/mol in this study using the Fe_2_O_3_-CNCs (of aspect ratio ~13 and with incorporated ~35 wt.% of Fe_2_O_3_ nanoparticles) as structural directing agents. It is noteworthy to mention that the energy of the intermediate state of bending of the graphene sheets to form GNS will significantly depend upon the morphological, physico-chemical properties and functionalization of the structural directing agents as well as graphene sheets. Eventually, after simulation run of ~400 ps, a complete nanoscroll is formed with the encapsulated Fe_2_O_3_-CNC in its core with dimensions of length similar to the template. Interestingly, the simulated Fe_2_O_3_-NS have slightly larger diameter compared to the template which was also observed in the experimental studies (FESEM/TEM of both Fe_2_O_3_-NS-HR and Fe_2_O_3_-NS-LR) suggesting that the central hollow core of Fe_2_O_3_-NS will be slightly larger than template diameter. In experimental conditions, the CNCs are removed through a controlled calcination process which further helps in scrolling of graphene sheets into perfect nanoscrolls mimicking the morphological dimensions of template with confinement of catalytically active metallic nanoparticles in its core.

### Fabrication of graphene nanoscrolls: Encapsulated catalytically active nanoparticles

**Figure 5:**
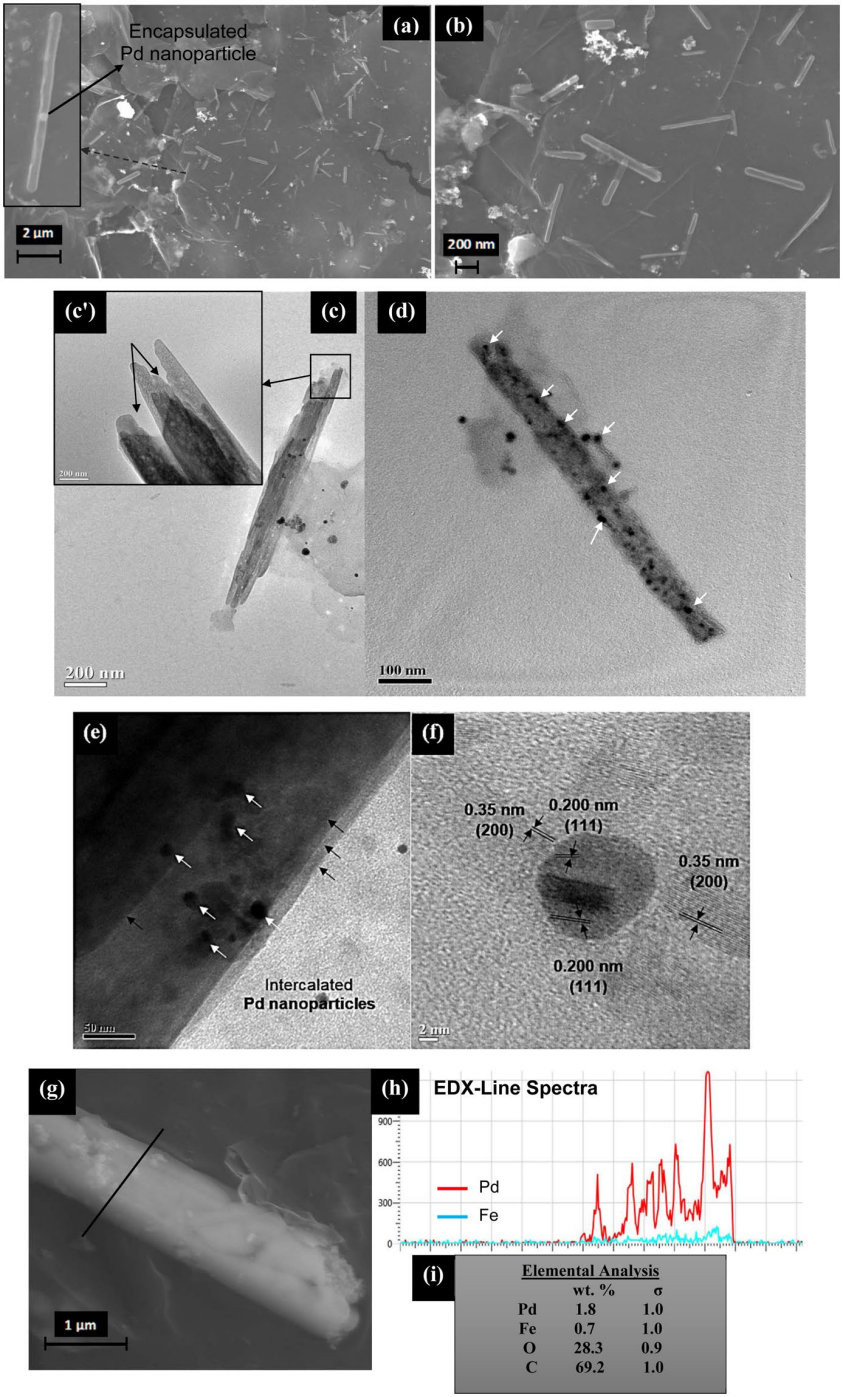
(**a**) FESEM micrographs of the fabricated Pd-Fe_2_O_3_-NS (with the inset showing a single GNS with the encapsulated Pd nanoparticle in its centre), (**b**) Pd-Fe_2_O_3_-NS FESEM micrograph at higher resolution, (**c**) and (**d**) TEM micrographs of the Pd-Fe_2_O_3_-NS with the encapsulated Pd nanoparticles in it (marked with white arrows) (**c’**) Inset displays the magnified open end of the Pd-Fe_2_O_3_-NS (marked with rectangular box), (**e**) TEM micrograph of a edge of Pd-Fe_2_O_3_-NS which shows the presence of multiple layer of graphene sheets rolled (marked with black arrows) with the Pd nanoparticles (marked in white arrows) encapsulated in its core and surface. (**f**) HRTEM micrographs of Pd-Fe_2_O_3_-NS illustrating the presence of lattice fringes of Pd nanoparticles and graphene, (**g**) and (**h**) FESEM-EDX line-spectra of the selected region (marked in black line) showing the distribution of the Pd and Fe elements on the Pd-Fe_2_O_3_-NS and (**i**) compositional analysis of the elements in the Pd-Fe_2_O_3_-NS.

Alongwith the tunable dimensions of GNS, the main objective of this study is to introduce different types of catalytically active metal precursors in its core which widens application of GNS in various sectors. Pd-Fe_2_O_3_-CNC and Pt-Fe_2_O_3_-CNC with high magnetic moment (~26.4 and 19.4 emu/g respectively) (Fig. S6(a–i)) and distinct rod/fiber-like morphology are used as templates for fabrication of GNS (as discussed in Supporting Information, Part II). In earlier reported literature^26^, attempt to incorporate palladium oxide nanoparticles in GNS core didn’t show any rolling behaviour. This is probably due to the absence of magnetic behaviour as well as structural directing templates which prevents graphene sheets to overcome the threshold energy required to initiate scrolling phenomenon. It has been observed through simulation studies that the separation distance between graphene and adsorbed nanoparticles is a key factor in initiating the scrolling process which is governed by vdW force of interaction^26^. During covalent functionalization of Pt/Pd-Fe_2_O_3_-CNC with graphene sheets through epoxidation process is expected to bring the two materials in close proximity which helps in overcoming the energy barrier, required for initiating the scrolling process. From morphological analysis, dimensions of Pd-Fe_2_O_3_-NS are of length ~710 ± 110 nm and diameter ~70 ± 22 nm, respectively (Fig. 5(a–d)). The distinct rod-like morphology of CNCs is prevalent in Pd-Fe_2_O_3_-NS even after its removal through calcination alongwith its comparable dimensions, suggests that the morphology of structural template is the governing factor in tuning the length of nanoscrolls. TEM micrographs (Fig. 5(d and e)) shows uniformly distributed monodisperse Pd nanoparticles (in size range of 10 ± 2 nm) along the length of nanoscrolls (marked with white arrows) with the presence of multiple-layered graphene scrolled (marked in black arrows) which have confined Pd nanoparticles in its core as well as inter-galleries. Interestingly, HRTEM micrographs (Fig. 5(f)) shows the presence of cross-over lattice fringes of ~0.350 and 0.200 nm corresponding to (200) and (111) crystal planes for graphene and Pd nanoparticles respectively. Further, line EDX spectra confirms the presence of both Pd and Fe elements with composition of ~1.8 wt.% and ~0.7 wt.% respectively, which suggests that utilization of magneto-responsive CNCs developed in this study acts as an unique template for introduction of catalytically active moieties in core of GNS.

**Figure 6:**
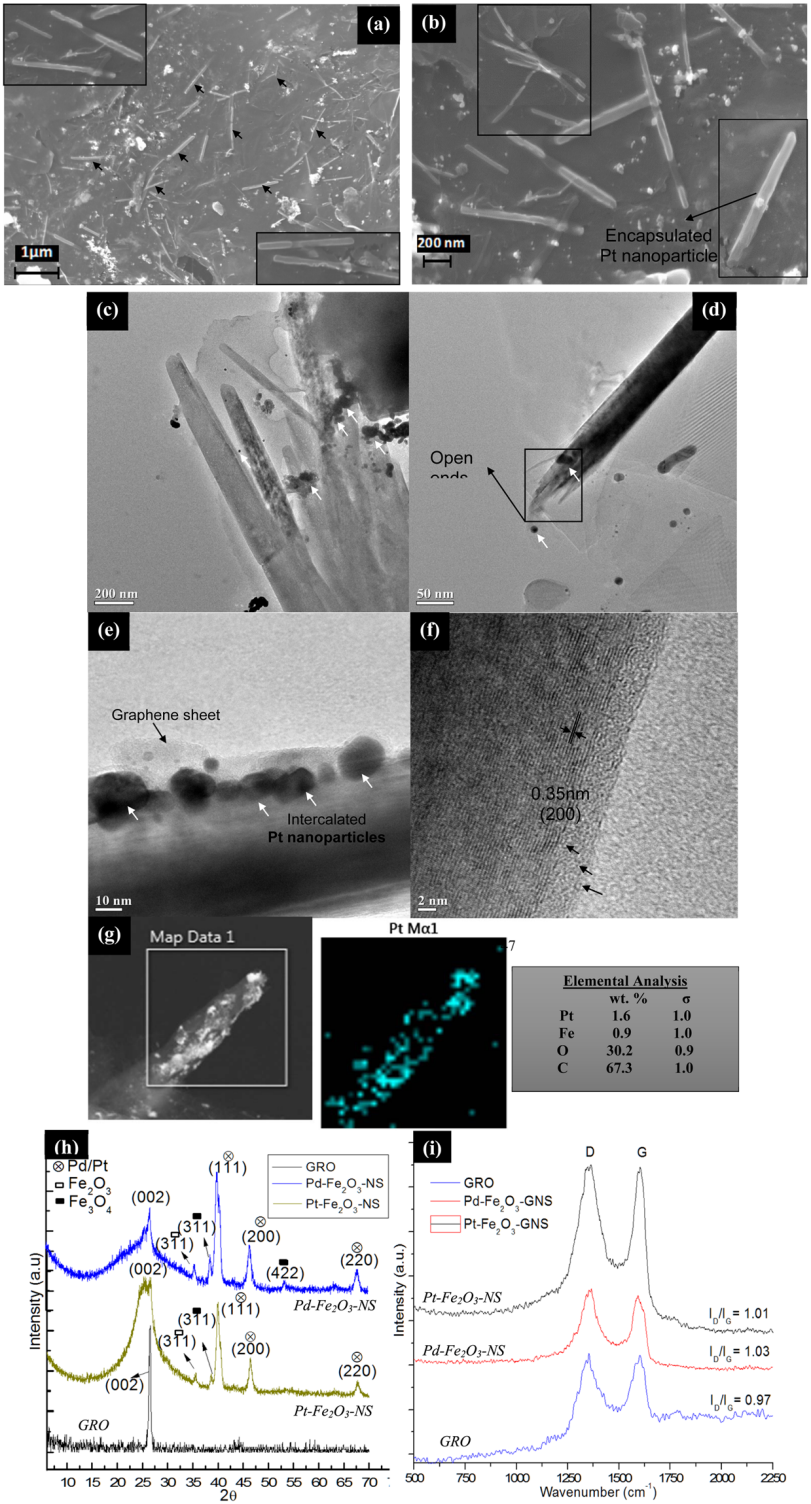
(**a**) FESEM micrographs of the fabricated Pt-Fe_2_O_3_-NS (marked with black arrows), (**b**) FESEM micrograph at higher resolution (with the inset showing a single GNS with the encapsulated Pt nanoparticle in its centre), (**c**) and (**d**) TEM micrographs of the Pt-Fe_2_O_3_-NS with the encapsulated Pt nanoparticles in it (marked with white arrows) (**d’**) Inset displays the magnified open end of the Pt-Fe_2_O_3_-NS (marked with rectangular box), (**e**) TEM micrograph of a edge of Pt-Fe_2_O_3_-NS which shows the graphene sheets rolled (marked with black arrows) with the Pt nanoparticles (marked in white arrows) encapsulated in its core and surface. (**f**) HRTEM micrographs of Pd-Fe_2_O_3_-NS illustrating the presence of lattice fringes of graphene, (**g**) and (**g’**) FESEM-EDX mapping of the selected region (marked in white box) showing the distribution of the Pt element on Pt-Fe_2_O_3_-NS and (**h**) compositional analysis of the elements in the Pt-Fe_2_O_3_-NS. (**h**) XRD diffractograms and (**i**) Raman spectroscopy of the Pt and Pd-Fe_2_O_3_-NS fabricated using catalytically active Pt-Fe_2_O_3_-CNCs and Pd-Fe_2_O_3_-CNCs as the structural directing templates.

To evaluate the robustness of Fe_2_O_3_-CNCs as structural directing agents for GNS formation, this study was further extended to encapsulate other forms of catalytically active Pt nanoparticles at lower loading fractions and evaluate the changes in its physico-chemical properties. Morphological analysis shows Pt-Fe_2_O_3_-NS are of length ~810 ± 90 nm and diameter ~95 ± 20 nm respectively, which is comparable with length of Fe_2_O_3_-NS with slight decrease in the diameter (Fig. 6(a–c)). It is expected that shrinkage in diameter of Pd/Pt-Fe_2_O_3_-NS might occur during calcination process when CNCs are removed from GNS core leaving behind Fe_2_O_3_/Pt-Pd nanoparticles while self-scrolling of graphene sheets due to inter-particle van der Waals force of attraction^3^ resulting in reduction of diameter. TEM micrographs displays bunch of hollow Pt-Fe_2_O_3_-NS with open ends and intercalated Pt nanoparticles (in size range of 11 ± 1 nm) distributed on its surface with thin layer of graphene sheets rolled on it (Fig. 6(d,e)), as confirmed from lattice spacing of ~0.35 nm representing (200) plane of graphene (Fig. 6(f)). Further, EDX-map of Pt-Fe_2_O_3_-NS shows uniform distribution of Pt nanoparticles with composition of ~1.6 wt.% confined in its core. Therefore, through strategic modifications of CNCs various types of catalytically active metallic nanoparticles can be confined in core of GNS with controlled loading fractions depending upon the desired catalytic activity required for targeted applications.

XRD diffractograms of Pd-Fe_2_O_3_-NS and Pt-Fe_2_O_3_-NS (Fig. 6(h)) shows broadening of peak at 2θ = 26.6° (corresponds to (002) planes of graphene) compared to sharp peak for GRO. Further, for Pd-Fe_2_O_3_-NS peak at 2θ = 26.6° shifts to 25.5° which is possibly due to scrolling of graphene sheets with different size of intercalated Pd/ Pt nanoparticles. Such alteration of diffraction peak of GNS(002) was also observed by Xu *et al*.^27^ on intercalation of different solvents followed by its swelling and drying. Pd-Fe_2_O_3_-NS, shows peaks at 2θ = 40.0°, 46.5° and 67.8° representing (111),(200) and (220) crystal planes of Pd nanoparticles^28^, peaks at 2θ = 35.5° corresponds to (311) crystal of Fe_2_O_3_ nanoparticles and low intensity peaks at 2θ = 38.6° and 53.4° designates (311) and (422) crystal planes of Fe_3_O_4_ nanoparticles. Similarly, Pt-Fe_2_O_3_-NS shows peaks at 2θ = 39.9°, 46.3° and 67.9° representing (111), (200) and (220) crystal planes of Pt nanoparticles^29^, peaks at 2θ = 35.6° corresponds to (311) crystal of Fe_2_O_3_ nanoparticles and a small peak at 2θ = 38.6° designates (311) crystal planes of Fe_3_O_4_ nanoparticles. The absence of any peak corresponding to CNCs crystallites suggests its complete removal, however, diffractograms shows presence of small intensity peaks of Fe_3_O_4_ nanoparticles which have plausibly formed during calcination process. Investigations of crystallites sizes of Pd/Pt nanoparticles (using Scherrer’s equation) encapsulated within GNS are ~8.6 nm and 10.7 nm respectively which comparatively matches with dimensions measured from TEM micrographs. This suggests that Pd/Pt nanoparticles adsorbed on CNCs can be effectively transferred to inner core of GNS without any significant agglomeration or accumulations of nanoparticles. From comparison of Raman spectra it was observed that I_D_/I_G_ ratio for GRO ~0.97, which increased to ~1.01 and 1.03 for Pd and Pt-Fe_2_O_3_-NS respectively (Fig. 6(i)). The increased I_D_/I_G_ ratio is attributed to formation of new defects as well as sp^3^ hybridization which might have occurred during epoxidation of graphene sheets with Pd/Pt-Fe_2_O_3_-CNC as well as calcination of CNCs to form hollow core. Moreover, increased broadness and intensity of G band in case of Pd/Pt-Fe_2_O_3_-NS is probably due to scrolling up of graphene sheets into multilayers which increases elastic strain on it. From crystallographic and spectroscopic studies, it was observed that the proposed methodology for fabrication of GNS efficiently utilizes CNCs as templates for encapsulation of wide variety of metallic nanoparticles inside GNS core depending upon targeted applications.

### Graphene nanoscrolls for hydrogen adsorption studies

**Figure 7:**
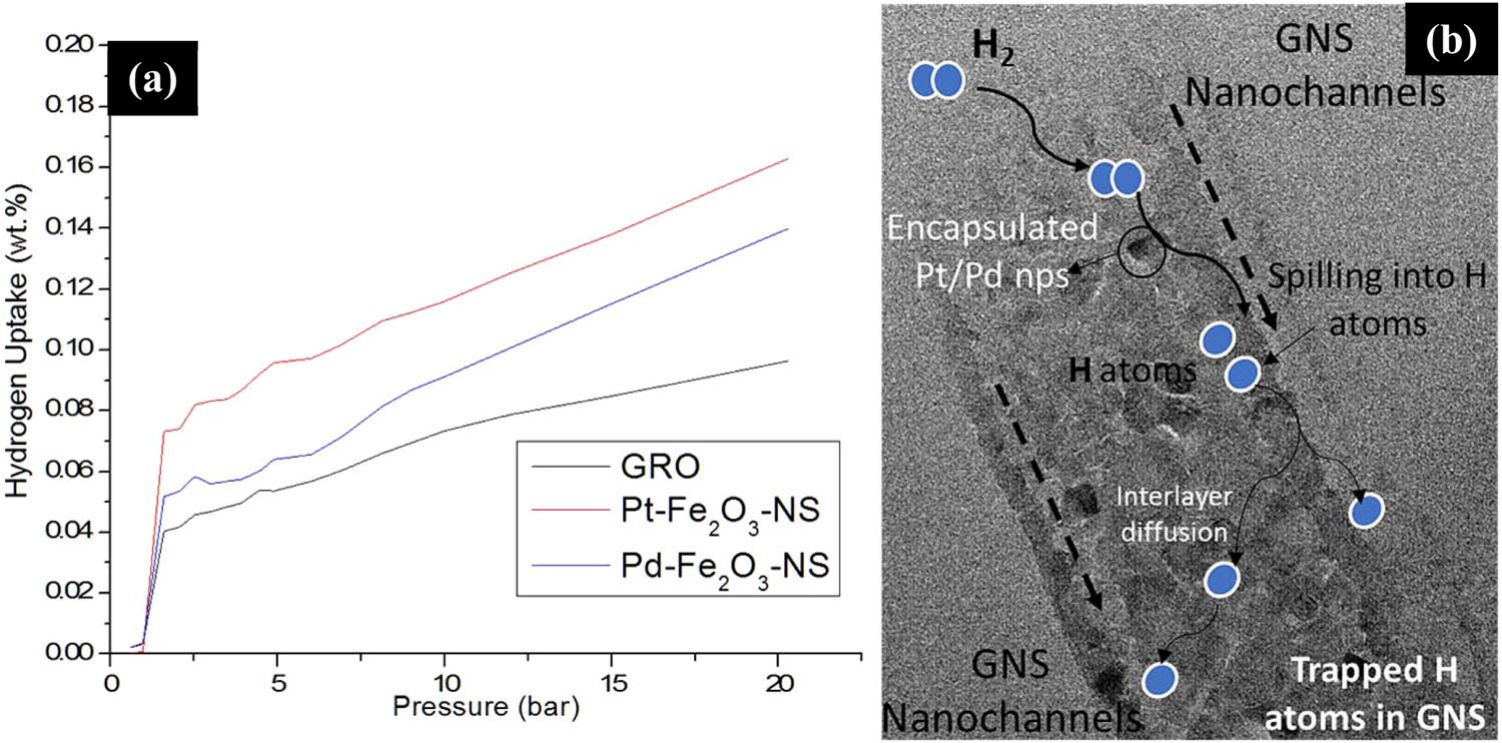
(**a**) Hydrogen adsorption isotherms of Pt-Fe_2_O_3_-NS and Pd-Fe_2_O_3_-NS in comparison with the initial graphene precursor, measured at 273 K and pressure range of 1–20 bar and (**b**) Plausible mechanism for H_2_ adsorption of GNS in which the encapsulated Pd/Pt nanoparticles primarily splits the H_2_ into H atoms, which undergoes inter-layer diffusion and gets embedded in the GNS nanochannels through physio-sorption or chemisorption routes resulting in enhanced H_2_ storage capacity.

The fabricated GNS have nanoscale hollow cylindrical channels doped with transition metal precursors such as Pd and Pt nanoparticles which is expected to provide a favourable route for hydrogen (H_2_) transport and storage. The H_2_ uptake for Pt-Fe_2_O_3_-NS and Pd-Fe_2_O_3_-NS are found to be ~0.17 and ~0.14 wt.% respectively (measured at 273 K and 20 bar) which is improved by ~112% in comparison to GRO (Fig. 7(a)). Most of the studies reported in literature on H_2_ adsorption of GNS are based on MD simulations, with a recent study showing H_2_ uptake of ~7 wt% at 77 K, 25 bar and ~0.4 wt% at 273 K, 25 bar pressure^8^, which is comparable to our experimental results. Interestingly, even with very low surface area of Pt-Fe_2_O_3_-NS and Pd-Fe_2_O_3_-NS (20.5 and 21.3 m²/g respectively) and lower fractions of encapsulated Pt/Pd nanoparticles (~1 wt%), significant improvement in H_2_ capacity is observed. This is probably due to the presence of encapsulated Pt/Pd nanoparticles which could dissociate H_2_ into H atoms through spill over mechanism^30^ followed by its entrapment in the GNS nanochannels through physio or chemisorption with unsaturated graphene layers (detailed mechanism explained in Fig. 7(b)). Therefore, it could be concluded that GNS with confinement of Pd/Pt nanoparticles (even at lower loadings) through the utilization of biobased nanomaterial as template, provides a novel approach to develop H_2_ storage devices operational at relatively feasible conditions (low pressure ~20 bar and 273 K).

### Electrochemical performance of graphene nanoscrolls as supercapacitors

**Figure 8:**
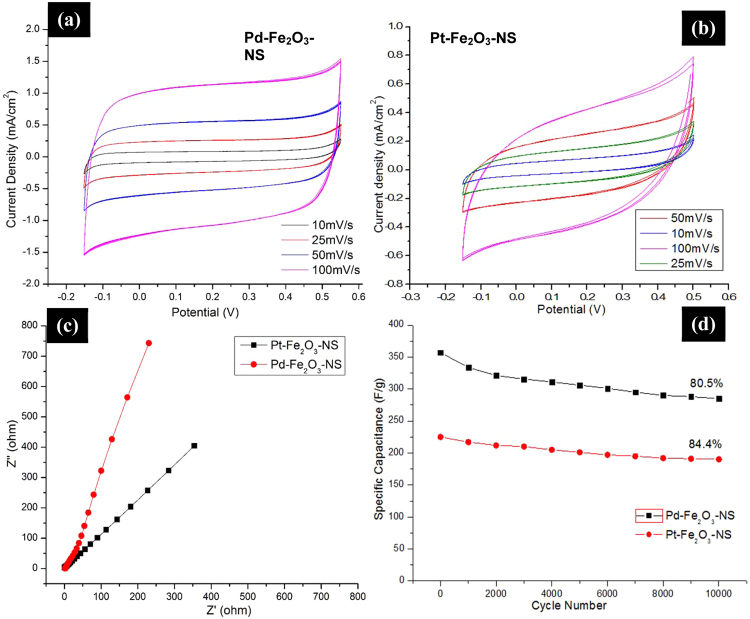
Cyclic Voltammograms of (**a**) Pt-Fe_2_O_3_-NS and (**b**) Pd-Fe_2_O_3_-NS measured at a scan rate of 10–100 mV/s, (**c**) Electrochemical impedance spectroscopy (EIS) or Nyquist plot for Pt-Fe_2_O_3_-NS and Pd-Fe_2_O_3_-NS, and (**d**) cyclic stability tests of Pt-Fe_2_O_3_-NS and Pd-Fe_2_O_3_-NS measured at current density of 1 A/g.

The scrolled topology of GNS favours the formation of electric double layer capacitors (EDLCs) alongwith the encapsulated metallic nanoparticles causes reversible faradaic redox reactions on its surface (electrode/electrolyte) introducing pseudocapacaitance behaviour, both of which is expected to act synergistically for enhancement of supercapacitance behaviour. Figure 8(a and b), CV curves of Pt-Fe_2_O_3_-NS and Pd-Fe_2_O_3_-NS measured at scan rate of 10–100 mV/s, shows asymmetric and quasi-rectangular shape which suggest that the nanochannels present in GNS provides increased charge propagation resulting in improved EDLC properties. With the increased scan rate, absence of any redox peaks or distortions in CV loops suggests its high capacitance retention capacity^31^. The specific capacitance of GNS is calculated from CV curves using equation, ${C}_{sp}=\int Idv/{\rm{\Delta }}V.v.m$, where *C*_*sp*_ is specific capacitance (F/g), *I* is average current (A), Δ*V* $\triangle {\rm{V}}$ is potential window, *v* is scan rate and *m* is mass of electroactive material^32^. The specific capacitance of Pd-Fe_2_O_3_-NS, Pt-Fe_2_O_3_-NS and Fe_2_O_3_-NS are calculated as 357, 223 and 152 F/g (at scan rate of 100 mV/s) respectively (Fig. S7(a)), which is significantly higher than the graphene or graphene based scrolls reported in literature^12,33^. Figure 8(c), shows the Nyquist plot with vertical slope at lower frequencies and negligible equivalent series resistance at higher frequencies (similar to earlier observations for GNS^12^), representing ideal capacitive behaviour of the material. The higher *C*_*sp*_ values for Pd-Fe_2_O_3_-NS in comparison to Pt-Fe_2_O_3_-NS is probably due to lowered defects and increased scrolling density which provides efficient pathways for diffusion of ions^34^, as evident from the Raman and XRD studies discussed in earlier section. The galvanostatic charge-discharge curves of Pt-Fe_2_O_3_-NS and Pd-Fe_2_O_3_-NS measured at a constant current density of 1 mA/cm^2^ are depicted in Fig. S7(b). All the GNS samples exhibited the formation of the triangular shapes with the absence of “IR drop” which suggests their supercapacitive behavior with low internal resistance of electrodes and improved electrode/electrolyte interface, however, shows variation in their charge/discharge time capabilities. The higher charge/discharge time for Pd-Fe_2_O_3_-NS, suggests that due to presence of lower defects, higher surface area and improved scrolling density resulted in the efficient transfer of ions into its nanochannels with the formation of strong EDL layers, which also resulted in significant improvement in performance of supercapacitors.

Further, long term cyclic durability tests retains specific capacitance by ~80.5 and 84.4% after 10000 cycles (charging-discharging between −0.15 to 0.55 V and −0.2 to 0.5 V for Pd-Fe_2_O_3_-NS and Pt-Fe_2_O_3_-NS respectively measured at 1 A/g) (Fig. 8(d)) which shows promising characteristics for practical applications of Pt/Pd-Fe_2_O_3_-NS as electrochemical charge storage devices. The higher retention of specific capacitance (compared to ~76.9% in case of Fe_2_O_3_-NS as shown in Fig. S7(c)) suggests that the nanochannels present in Pt/Pd-Fe_2_O_3_-NS are stable enough providing efficient routes for diffusion of ions alongwith the entrapped catalytically active moieties which are site for multi-electron reversible redox reactions. Therefore, it is envisaged that topological feature of GNS in terms of scrolling density, inter-layer spacings and morphological dimensions can be remotely tuned to enhance EDLC properties followed by encapsulation of variety of catalytically active metallic species in their inter-galleries to induce pseudo-capacitive properties, both of which needs to be optimized such that they could synergistically maximize the supercapacitive behaviour for fabrication of high performance charge storage devices.

## Discussions

CNCs derived from the renewable resources, acts as a unique template for fabrication of GNS with tunable morphologies ranging from micron to nanoscale dimensions (in length), which are otherwise difficult to control by traditional routes of GNS processing. Through strategic surface modification of CNCs, catalytically active metallic nanoparticles can be incorporated in GNS core (with desired loadings), tunable inter-layer spacings and scrolling density depending upon the targeted applications. From molecular simulation studies, it was observed that formation of GNS using Fe_2_O_3_-CNCs as templates is a thermodynamically favourable process which undergoes self-assembly in the presence of van der Waals forces and π-π interactions that are found to depend on several parameters such as density of maghemite nanoparticles, magnetic moment, morphology and dimension of CNCs. The metal nanoparticle encapsulated GNS with unique structural properties and physico-chemical characteristics shows potential applications in fabrication of enhanced hydrogen storage devices and supercapacitors possessing long-term cyclic stability (of ~10000 cycles). The utilization of biobased CNC precursors for fabrication of GNS with tunable morphology, controlled dimensions alongwith the encapsulation of metallic nanoparticles, opens up plethora of multifunctional applications of GNS in diverse platforms with promising commercial opportunities.

## Materials and Methods

### Materials

Expandable graphite (EG, 99.9%) purchased from Asbury Carbons (USA) was used for fabrication of graphene. The chemical reagents such as sodium borohydride (NaBH_4_) (>99% purity), hydrogen peroxide (30%), ferric (III) chloride (>96%), 4-nitrophenol (4-NP), sodium hydroxide (NaOH) (>97%), sodium hypochlorite (4%), and sulphuric acid (>99%), (Analytical Grade (AR)) used in the study were obtained from SISCO Research laboratories (SRL Chemicals, India). Bamboo pulp, was selected as the initial cellulosic precursor for the fabrication of CNCs, which was received from Hindustan Paper Corporation Limited (HPCL, Assam, India). The platinum (PtCl_2_) (99.9%) and palladium chloride (PdCl_2_) (99%) salts used in this study were purchased from Sigma Aldrich, India.

### Fabrication of Magnetic cellulose nanocrystals (Fe_2_O_3_-CNCs)

CNCs were fabricated from the bamboo pulp through soda pulping cum bleaching pretreatments (to extract the purified cellulose) followed by stringent sulphuric acid (64 wt%) hydrolysis, as per our earlier reported studies^35^. FeCl_3_ (2.0 g), was selected as initial iron precursor and dispersed into CNC suspension (1.0 g in 50 ml of ~1 wt% NaOH solution) through sonication at an amplitude of ~30% for 5 min. The dispersed iron salt-CNC suspensions was stirred for ~3 hours at 80 °C purged under inert nitrogen gas flow for ~3 hours to remove the trace amount of oxygen, as per the protocol reported in our earlier studies^23,36^. The precipitation of iron nanoparticles was carried out by dropwise addition of the reducing agent, sodium borohydride (~50 mg/ml) under vigorous stirring (~500 rpm) at a temperature of ~90 °C. After ~6 hours, the iron salt-CNC suspension turned black suggesting the completion of reaction and was separated using permanent magnets followed by washing it with ethanol (for three times) and drying (at 50 °C in vacuum oven) to obtain the powdered form of Fe_2_O_3_-CNCs. Fe_2_O_3_-CNCs with higher aspect ratio were fabricated using CNCs derived from filter paper as initial cellulosic precursor^19^ following similar protocol for precipitation of iron nanoparticles as mentioned above. The detailed data for its characterization is available in the Supporting Information (Part I).

### Fabrication of graphene nanoscrolls (GNS) using Fe_2_O_3_-CNCs as templates

Graphene was fabricated through thermal exfoliation of expandable graphite with the optimized conditions mentioned in our earlier study^37^. The obtained graphene (~500 mg) was dispersed into methanol followed by its sonication (at amplitude of ~30%) for duration of ~30 mins in presence of the hydrogen peroxide (~2wt.%) as per the literature^38^ to introduce epoxy and hydroxyl functionalities.

Epoxidation of Fe_2_O_3_-CNCs was carried out through reaction of Fe_2_O_3_-CNCs (~50 mg dispersed in ~20 ml of water through sonication at an amplitude of ~30% for 5 min) with excess epichlorohydrin (~5 ml), at a temperature of ~85 °C for ~4 hours^39^. The epichlorohydrin modified Fe_2_O_3_-CNCs, was washed with water and ethanol (for ~3 times) to remove the unreacted components, followed by drying (at 50 °C in vacuum oven).

Fabrication of GNS using Fe_2_O_3_*-*CNCs as templates was carried out through reaction of epoxidized Fe_2_O_3_*-*CNCs with partially oxidized GRO (at a ratio of 1:1 dispersed in 50 ml methanol) at a temperature of 90 °C under reflux conditions which leads to covalent coupling between the two systems. The reaction was continued for ~6 hours followed by washing with ethanol (~3 times) and then subjected to controlled calcination. The calcination process was carried out in muffle furnace (samples placed in a silica crucible) with controlled temperature program of: 200 °C for 2 hours, 400 °C for 2 hours and 600 °C for 15 mins. Similar protocol was followed for fabrication of Fe_2_O_3_-NS with higher aspect ratio. The fabricated GNS are designated as Fe_2_O_3_-NS-HR and Fe_2_O_3_-NS-LR with high and low aspect ratio respectively, with the encapsulated iron oxide nanoparticles in core of GNS.

### Fabrication of platinum and palladium modified magnetic cellulose nanocrystals (Pt-Fe_2_O_3_-CNCs and Pd-Fe_2_O_3_-CNCs)

Platinum and palladium nanoparticles modified Fe_2_O_3_*-*CNCs were fabricated through precipitation of the platinum and palladium chloride salts using sodium borohydride as reducing agent under inert conditions. A homogenous dispersion of Fe_2_O_3_*-*CNCs (of low aspect ratio) was formed by dissolving PtCl_2_ and PdCl_2_ (~50 mg) salts (in slightly acidic water), through sonication (for ~5 min) followed by stirring at ~500 rpm for 3 hours at 80 °C which led to formation of light yellowish/ brownish coloration. The impregnated salts on the surface of Fe_2_O_3_*-*CNCs were precipitated through dropwise addition of NaBH_4_ solution under vigorous stirring (~500 rpm) at a temperature of ~90 °C for 4 hours which subsequently led to formation of grey-black coloration.

### Fabrication of graphene nanoscrolls using Pt-Fe_2_O_3_-CNCs and Pd-Fe_2_O_3_-CNCs as templates

Pt*-*Fe_2_O_3_*-*CNCs and Pd*-*Fe_2_O_3_*-*CNCs (~20 mg/ml in water) was epoxidized following the similar protocol as mentioned above through reaction with epichlorohydrin for ~6 hours at ~85 °C. The scrolling of graphene sheets was carried out through reaction of epoxidized Pt*-*Fe_2_O_3_*-*CNCs or Pd*-*Fe_2_O_3_*-*CNCs (~50 mg dispersed in methanol) with GRO (~50 mg) at a temperature of 90 °C under reflux conditions for ~6 hours. After the reaction is complete, samples were centrifuged to obtain the precipitate followed by its washing (with ethanol) and drying. The obtained dried samples were placed on a silica crucible and calcined in a muffle furnace to remove the CNCs from its inner core following the similar temperature program as mentioned above. The fabricated samples are designated as Pd*-*Fe_2_O_3_*-*NS and Pt*-*Fe_2_O_3_*-*NS, with the encapsulated Pt and Pd nanoparticles in GNS.

### Molecular dynamics simulations of graphene nanoscroll formation

Molecular dynamics (MD) simulation of the scrolling of graphene sheets onto Fe_2_O_3_-CNC as substrate was carried out using the Discover module of the Materials Studio 7.0 software package. In this work, we have used universal force field (UFF) to describe the interatomic interactions between the graphene sheet and Fe_2_O_3_*-*CNCs. The MD simulation was performed in the NVT ensemble at 298 K for a total simulation time of ~500 ps with a time step of 1 fs. The data were recorded every 1 ps to determine the changes in the trajectory of atoms. The Nosé-Hoover thermostat was used to maintain the temperature constant. Further, the van der Waals interactions between the graphene sheets and Fe_2_O_3_-CNC were calculated with a cut-off distance of 9.5 Å. The electrostatic interactions were calculated using the Ewald summation method with the same cut-off distance of 9.5 Å. It should be noted that geometry optimization was carried out on all the prepared structures namely the graphene sheet, Fe_2_O_3_-CNC, and Fe_2_O_3_-CNC/graphene system before starting the MD simulation.

### Instrumentation and characterizations

The powder X-ray diffraction (XRD) of fabricated nanoscrolls was recorded with D8 Advance diffractometer (Bruker, Germany) utilizing Cu-K_α_ radiation (λ = 0.1541 nm) as X-ray source (40 kV, 40 mA) measured at scan rate of 0.05° per 0.5 s in the 2θ range ~5–70°. The chemical functionalities in graphene nanoscrolls were analyzed by Raman spectroscopy using Horiba Jobin Vyon (LabRam HR, Japan) equipped with a 1 W, 1064 nm Nd:YAG diode-pumped laser at an excitation wavelength of 514 nm, exposure time of ~5secs and 1024 scans. The surface area was calculated through Brunauer–Emmett–Teller (BET) method based on nitrogen adsorption-desorption at 77 K using a Beckman Coulter surface area analyzer (Coulter SA 3100, USA). Prior to nitrogen physisorption, the nanoscrolls were degassed at 393 K for 2 hours to remove the adsorbed moisture. Morphological dimensions and structure of the fabricated nanoscrolls were characterized using TEM micrographs, high resolution TEM (HRTEM) and selected area electron diffraction (SAED) patterns. The uniformly dispersed graphene nanoscrolls suspensions (in methanol) were drop cast onto carbon-coated grids (Tedpell, USA) and vacuum dried overnight at 60 °C. Images were captured using JEOL JEM 2100 TEM at an accelerating voltage of 200 kV, and the average dimensions were determined (using Image J software). The graphene nanoscrolls samples were coated with gold sputtering unit and the morphology was characterized using Field Emission SEM (FESEM) (Zeiss, Germany, Model Sigma) at an accelerating voltage of 2–20 kV. The compositional analysis and mapping of the Pd and Pt nanoparticles in GNS were determined through energy dispersive X-ray analysis (EDX) (Oxford Instruments, UK) at an accelerating voltage of 20 kV using Aztec software.

### Hydrogen uptake measurements

Hydrogen uptake by Pd*-*Fe_2_O_3_*-*NS and Pt*-*Fe_2_O_3_*-*NS are measured over a varied pressure range using temperature programmed volumetric sorption apparatus (Model: iSorb-HP, Make: Quantachrome). Prior to analysis the samples were initially outgassed at 150 °C for 30 min in presence of inert gas flow. Subsequently, the hydrogen was allowed to adsorb on the samples at a constant temperature of ~273 K with increasing pressure from 0 to 20 bar. Similarly, desorption of absorbed hydrogen was allowed to occur with the declining pressure maintained at a constant temperature.

### Electrochemical performance and measurements

The working electrode was fabricated by dispersing GNS (~6 mg) into iso-propyl alcohol with addition of binder 5% Nafion solution, followed by coating on Toray carbon paper (1 cm^2^) and drying at 90 °C for overnight. The electrochemical measurements, cyclic voltammetry (CV) (at scan rate: 10–100 mV/s and voltage: 0.2 to 0.6 V), galvanostatic charge-discharge (GCD) and electrical impedance spectroscopy (EIS) (frequency range: 0.01 Hz-10 kHz) were measured using electrochemical workstation Metrohem Autolab PGSTAT 204 operated with a three electrode system, (reference electrode: Ag/AgCl/3 M KCl and counter electrode: platinum) in presence of 0.5 M KOH as electrolyte solution.

## Electronic supplementary material

Supplementary Information
