# Supplementary material for: Cellulose Nanocrystal Templated Graphene Nanoscrolls for High Performance Supercapacitors and Hydrogen Storage: An Experimental and Molecular Simulation Study
Source: Sci Rep. 2018 Mar 1;8:3886. doi: 10.1038/s41598-018-22123-0 (PMC5832814; doi:10.1038/s41598-018-22123-0)
Supplement: Supplementary file 1 — Supplementary Information [file 41598_2018_22123_MOESM1_ESM.doc]

Supporting Information

Cellulose Nanocrystal Templated Graphene Nanoscrolls for High Performance Supercapacitors and Hydrogen Storage: An Experimental and Molecular Simulation Study

Prodyut Dhar, Surendra Singh Gaur, Amit Kumar and Vimal Katiyar*

Department of Chemical Engineering, Indian Institute of Technology Guwahati, Guwahati, 781039, Assam, India.

*Corresponding author, email: [vkatiyar@iitg.ac.in](mailto:vkatiyar@iitg.ac.in)


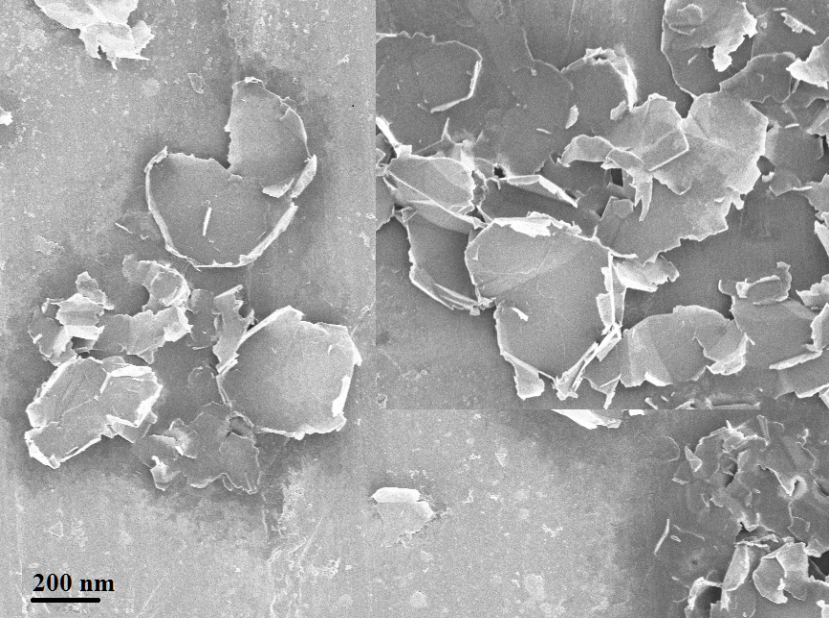


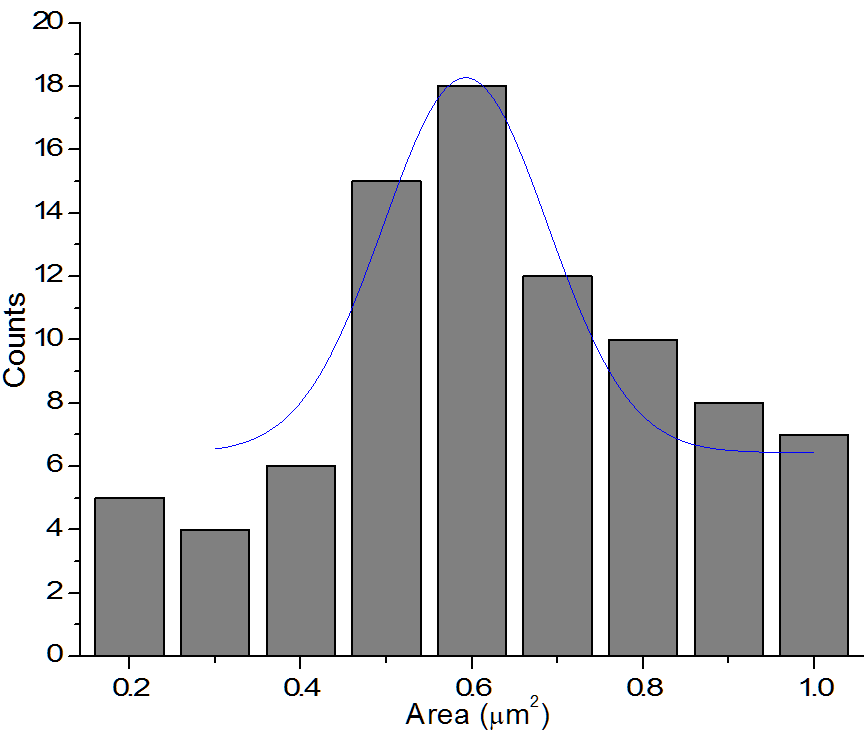

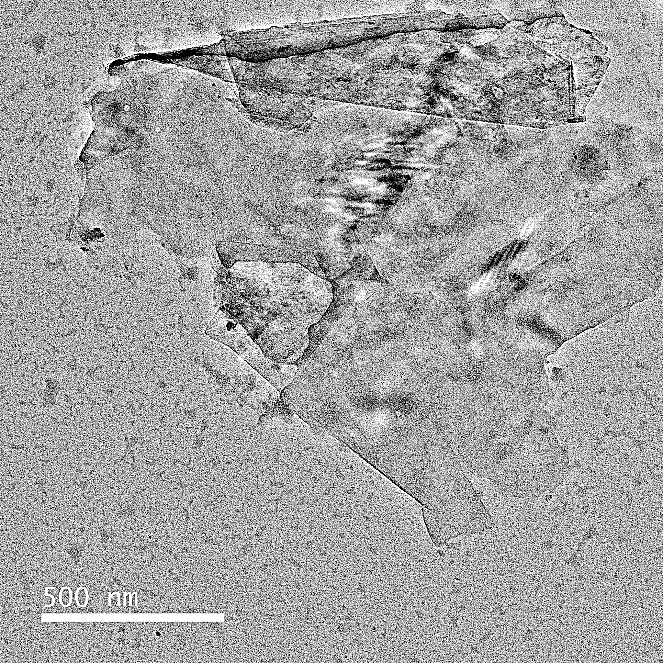


**(a)**

**(b)**


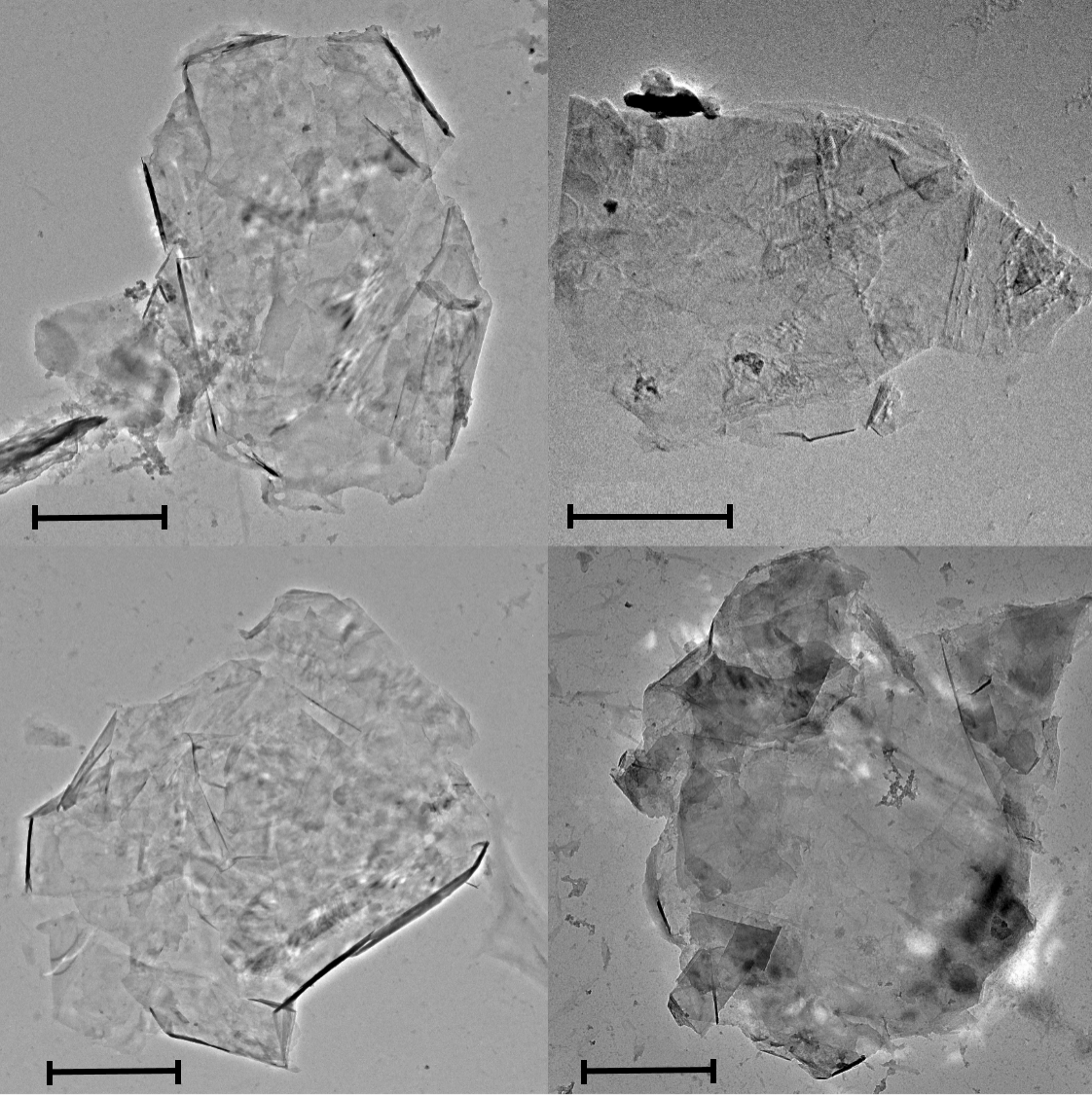

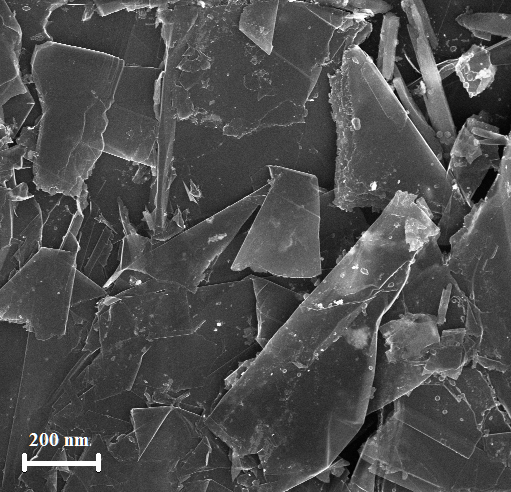


**(c)**

**(d)**

**(d')**

***Figure S1*:** *(a) TEM micrographs of the partially oxidized graphene (GRO) sheets, (b) histograms of area of the GRO sheets, (c) FESEM micrographs of the GRO sheets and (d)and (d') Collected TEM and FESEM micrographs of GRO sheets (scale bar: 500nm)*

***Part I:*** *Fabrication and characterization of iron oxide nanoparticles modified CNCs.*

CNCs fabricated from pretreated bamboo pulp after sulphuric acid hydrolysis was selected as template for the precipitation of Fe_2_O_3_ nanoparticles in presence of reducing agent. After the reduction reactions, smooth rod-like surface of CNCs are modified with spherical Fe_2_O_3_ nanoparticles. This led to alteration in its morphological dimensions with an average length of ~745±63nm and diameter of ~56±8nm, in which the distinct rod-like morphology of CNCs is prevalent with an aspect ratio ~13 (as shown in Fig. S2 (a) and (b)). The TEM micrographs shows that spherical Fe_2_O_3_ nanoparticles of size distribution ~20-32nm gets anchored onto CNCs (as shown in Fig. S2(c) and (c')) due to strong electrostatic interaction between the positively charged metal ions and negatively charged hydroxyl groups, as per our earlier reported studies. XRD diffractograms of Fe_2_O_3_-CNC, shows the presence of diffraction peaks at 2Ɵ=30.4°, 35.8°, 43.2°, 53.7°, 57.4° and 63.0° which corresponds to the crystal planes of Fe_2_O_3_ nanoparticles at (220), (311), (400), (422), (511) and (440) respectively ^1^. The small intensity peaks obtained at 2Ɵ=14.8°, 16.5° and 22.6° with (1̅10), (110) and (002) plane represents the cellulose I crystal structure of CNCs (as shown in Fig. S2 (d)), which remains unaltered during the precipitation process. The minimum crystallite size of Fe_2_O_3_ nanoparticles adsorbed onto CNCs are measured to be ~21nm (using Scherrer’s formulae corresponding to the diffraction peak at 2Ɵ=35.8°), which falls within the size range calculated from FESEM micrographs. Therefore, from crystallographic studies it can be confirmed that Fe_2_O_3_ nanoparticles of uniform size distribution can be adsorbed onto CNCs through simple single-step precipitation of iron salts in presence of reducing agents.

The fractions of Fe_2_O_3_ nanoparticles incorporated onto the surface of CNCs are determined through TGA and EDX analysis of the Fe_2_O_3_-CNC (as shown in Fig. S2 (e) & (f)). TGA thermograms shows that CNC undergoes slight weigh loss in range of ~100–200°C corresponding to the removal of moisture and sulphate groups present on the CNC surface followed by the complete degradation of cellulose in range of ~250–500°C. Incorporation of Fe_2_O_3_ nanoparticles onto the surface of CNCs, led to improved thermal stability of CNCs with the reduction in weight by only ~20-25% in the temperature range of 150-400°C. This is probably due to incorporation of Fe_2_O_3_ nanoparticles which generally have increased thermal stability that remains unaltered till ~600°C. The percentage fractions of Fe_2_O_3_ nanoparticles present on the surface of CNCs was determined from the difference in percentage weight losses of CNC and Fe_2_O_3_-CNC thermograms measured at ~500°C. From the thermogravimetric analysis, it was observed that about ~ 35 wt. % content of Fe_2_O_3_ nanoparticles are incorporated on the surface of CNCs. Subsequently, the compositional analysis of Fe_2_O_3_-CNC through EDX spectroscopy, also confirms the presence of ~ 38 wt. % of Fe alongwith ~37 wt. % of O and ~24 wt. % of C elements, respectively. Introduction of such high content of Fe is probably due to the improved interaction between the hydroxyl groups of CNCs with the iron salts (as discussed in earlier sections), which enhances the nucleating sites for growth of Fe_2_O_3_ nanoparticles during precipitation reactions^2^.

The surface modifications of CNCs with such high content of Fe_2_O_3_ nanoparticles results in the introduction of the magneto-responsive behaviour, which have been evaluated through VSM studies measured at 298K (as shown in Fig. S2 (g)). VSM curves shows that the Fe_2_O_3_-CNC have high magnetization values of ~43 emu/g due to the presence of high fractions of Fe_2_O_3_ nanoparticles (~35 wt. %) compactly adsorbed onto the surface of CNCs which leads to introduction of ferromagnetic behaviour. Fabrication of such catalytically active ferromagnetic nanocrystals from naturally derived biomass resources alongwith the interesting properties of non-toxicity and biocompatibility finds potential applications in fields related to biomedical engineering and healthcare products. To determine the effect of high aspect ratio Fe_2_O_3_-CNC on the formation of GNS, Fe_2_O_3_-CNC with morphological dimension of length~2.8±0.8μm and diametre~82±18nm with aspect ratio of~35 have also been fabricated (as shown in Fig. S3), using the CNCs derived from the filter paper as cellulosic precursor. It has been observed that the presence of high magnetic moment of maghemite nanoparticles alongwith the distinct rod-like morphology of Fe_2_O_3_-CNC provides a unique opportunity to scroll up the graphene sheets on its surface as structural template, which have been discussed in subsequent sections.


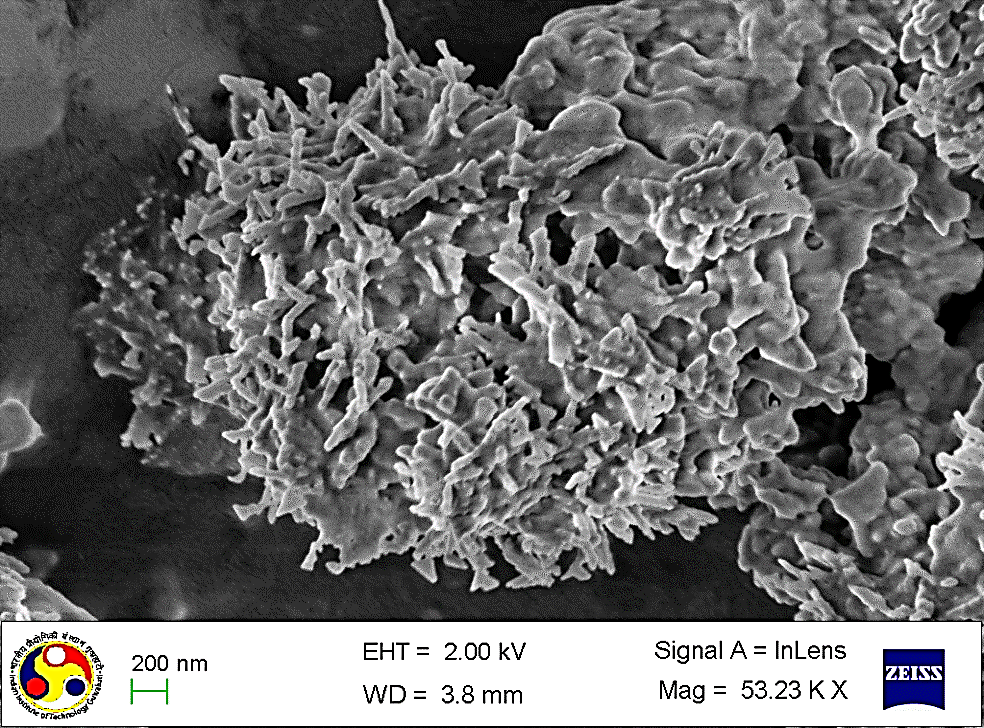


**(a)**


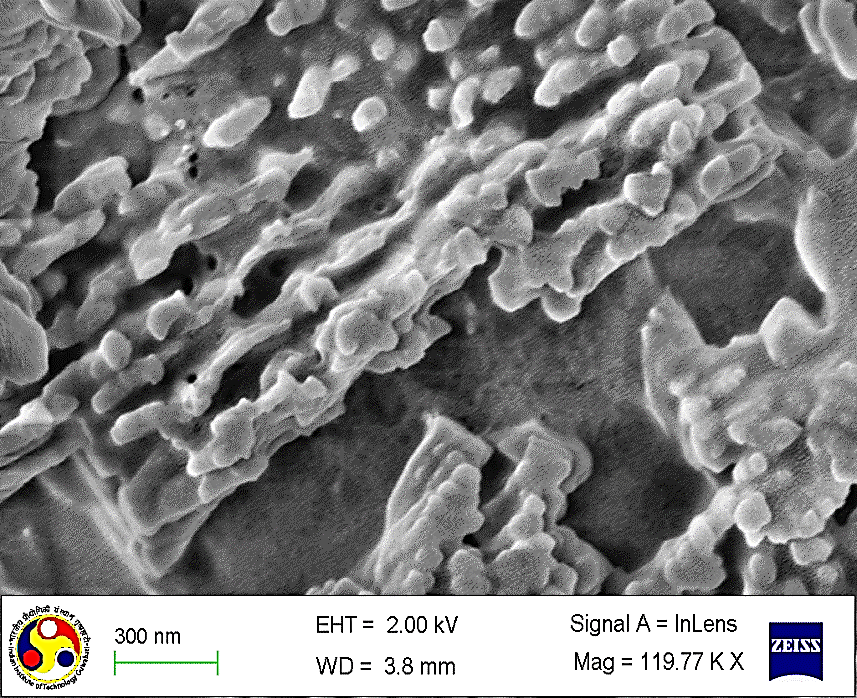


**(b)**


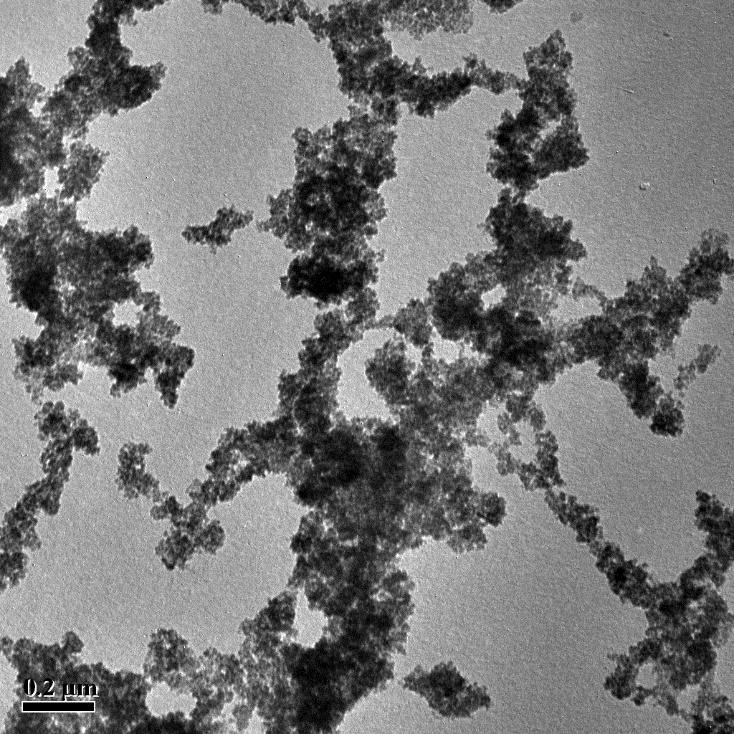


**(c)**


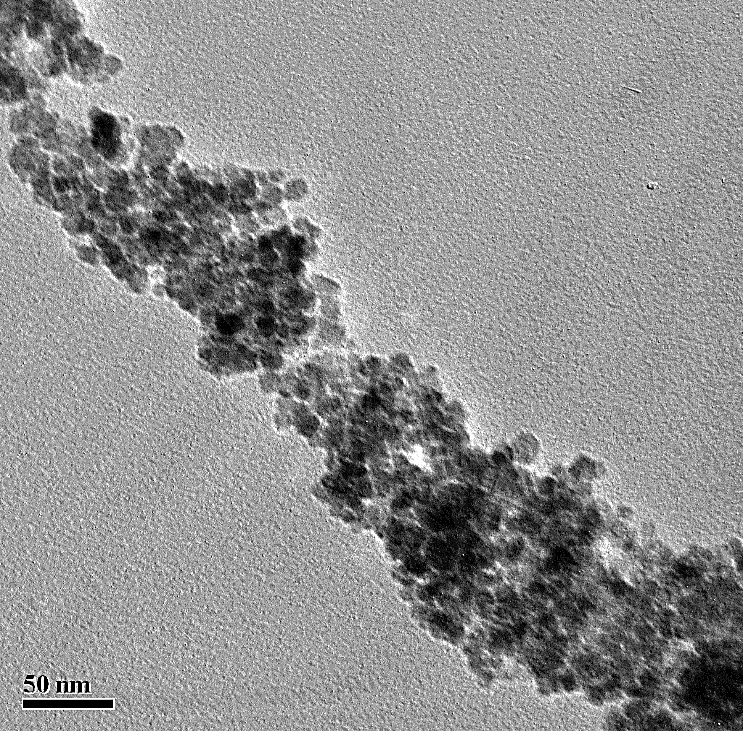


**(c')**


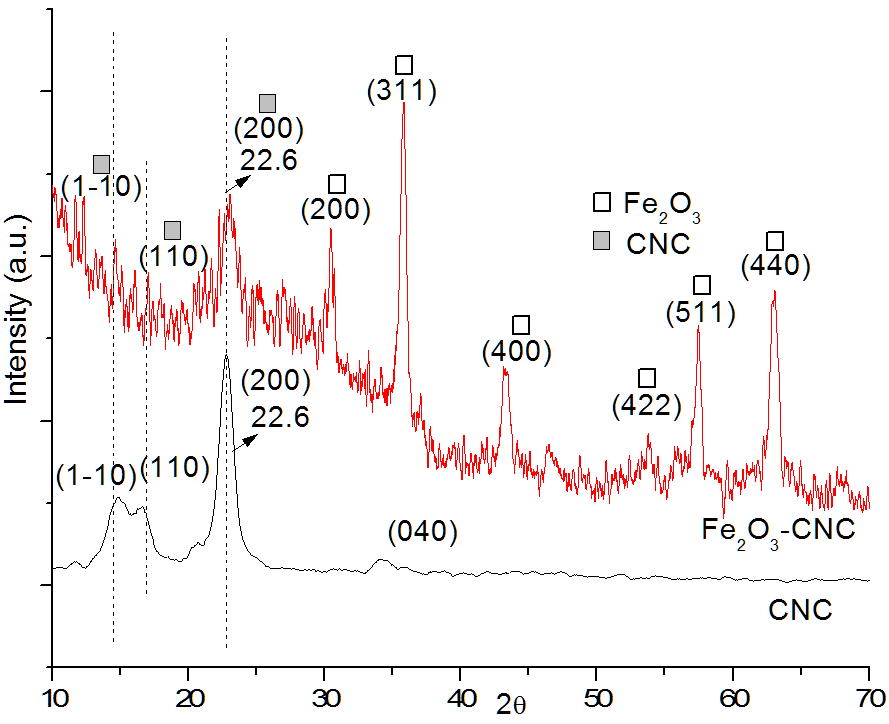


**(d)**


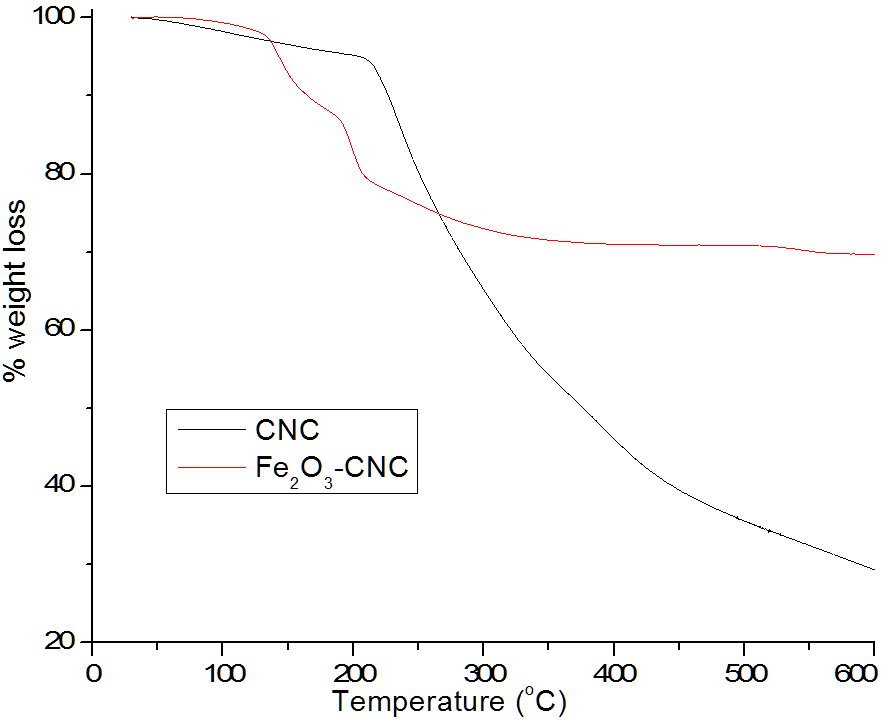


**(e)**


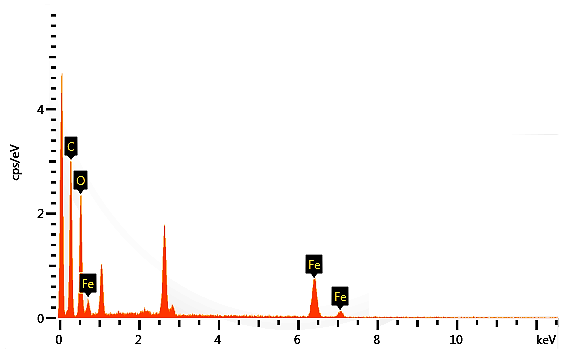


**Elemental Analysis**

**wt. % σ**

**Fe 38.2 1.0**

**O 37.3 0.9**

**C 24.5 1.0**

**(f)**

keV


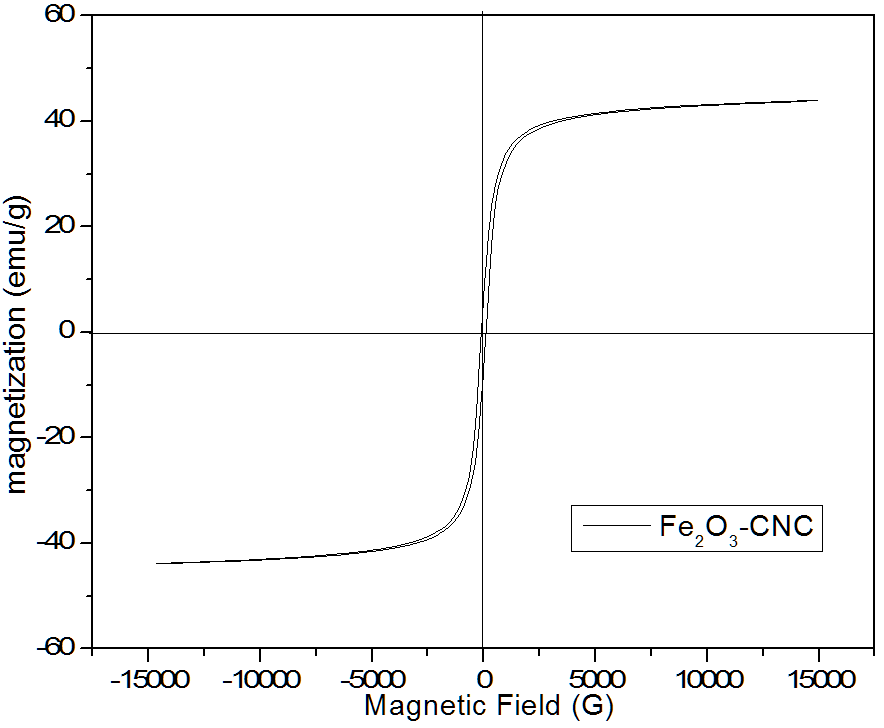


**(g)**

***Figure S2*:** *FESEM micrographs of the fabricated (a) Fe_2_O_3_-CNC through a single step precipitation method, (b) high resolution micrographs of Fe_2_O_3_-CNC (at a resolution of ~100KX) and (c) and (c')TEM micrographs of Fe_2_O_3_-CNC which shows the presence of spherical Fe_2_O_3_ nanoparticles adsorbed onto CNCs, (d*) *XRD patterns of Fe_2_O_3_-CNC with the crystal planes of Fe_2_O_3_ and CNC marked in white and black squares respectively*, *(e) TGA profiles for the Fe_2_O_3_-CNC and CNCs respectively, (f) EDX spectra of Fe_2_O_3_-CNC with the respective composition of elements (in wt.%) shown in the table (inset) and (g) Magnetic hysteresis loop for the fabricated Fe_2_O_3_-CNC measured at 298K.*

***Figure S3*:** *FESEM micrographs of the Fe_2_O_3_-CNC fabricated with high aspect ratio CNCs as precursor* (*derived from filter paper through sulphuric acid hydrolysis as mentioned in* ^3^)*.*


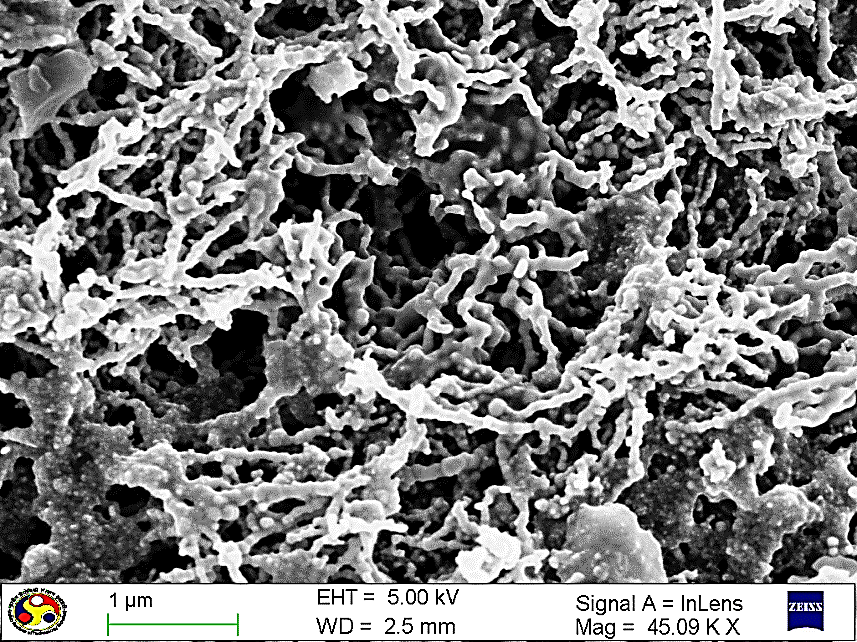

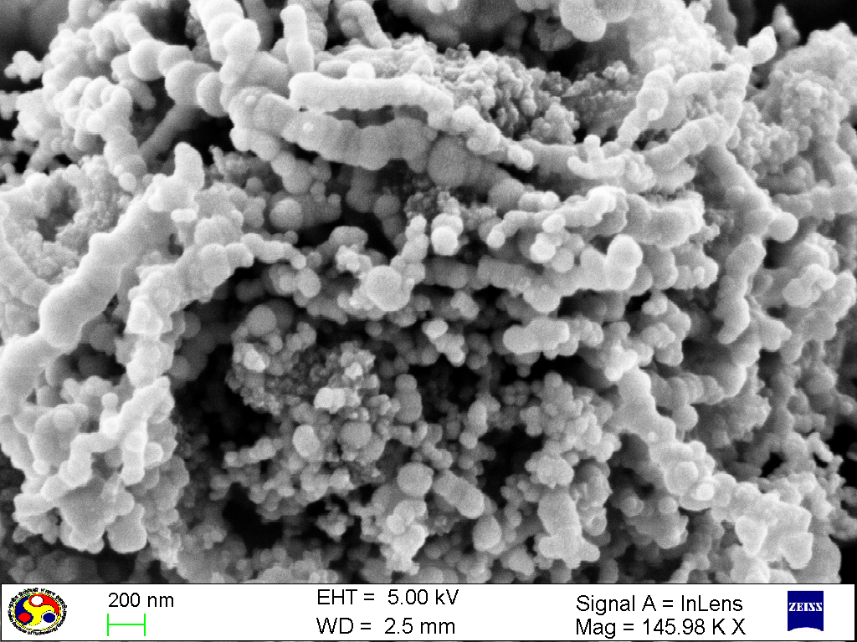

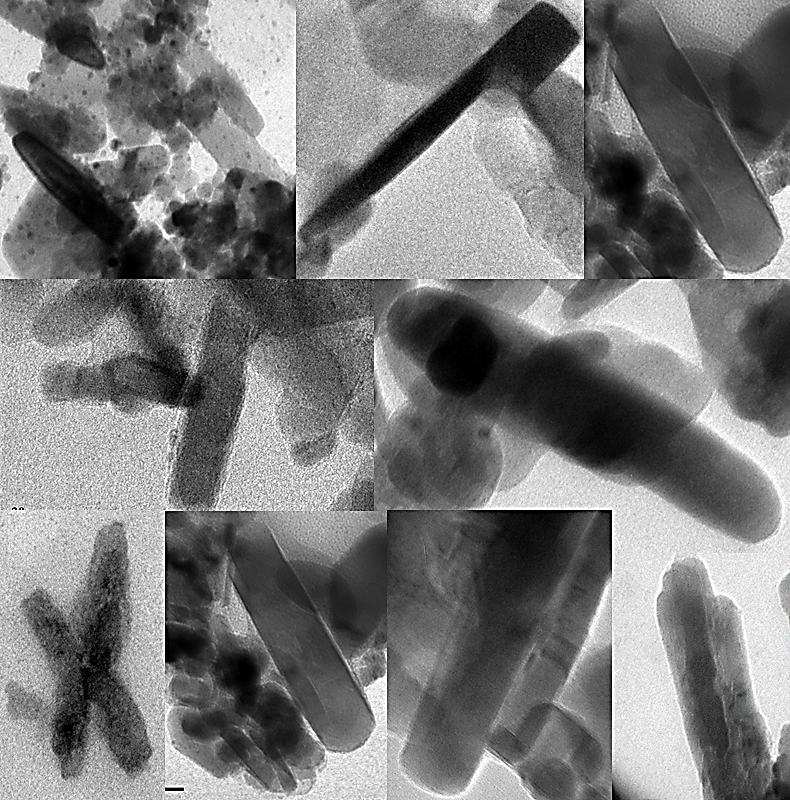


**(a)**


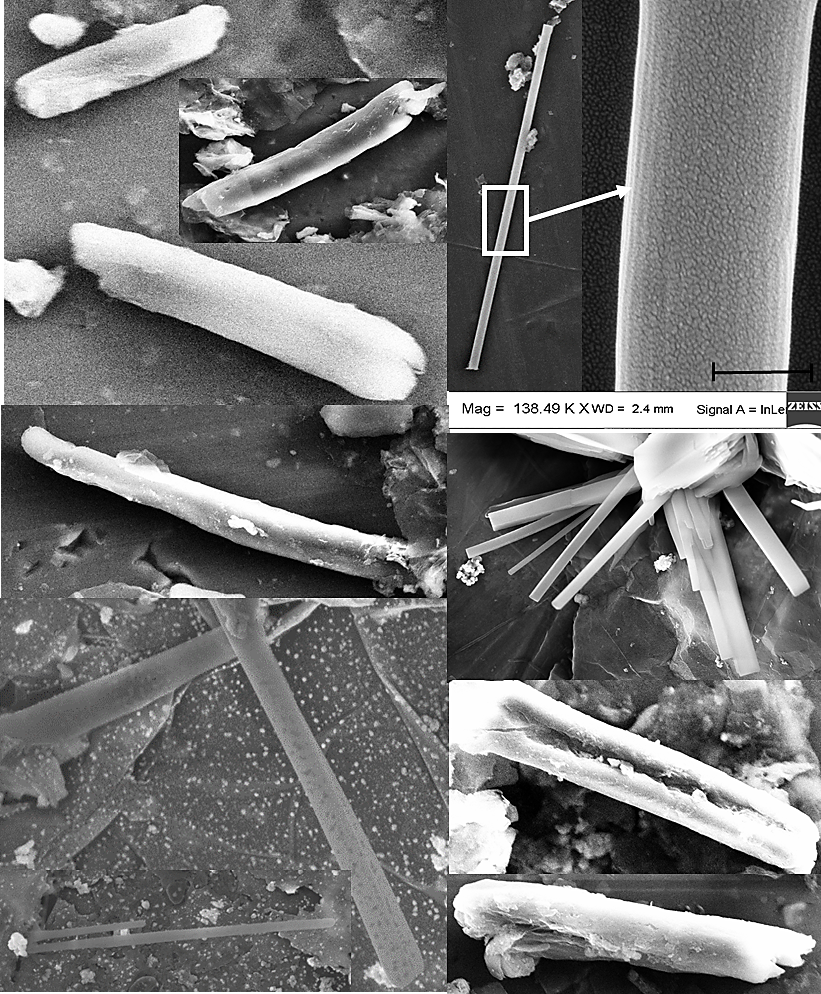


**200 nm**

**(b)**

***Figure S4:*** *(a)* *Collected TEM micrographs of the**Fe_2_O_3_-NS fabricated using low aspect ratio Fe_2_O_3_-CNCs as initial precursor from several TEM grids with morphological dimensions in similar range (scale bar: 100 nm) and (b) Collected FESEM micrographs of the* *Fe_2_O_3_-NS fabricated using high aspect ratio Fe_2_O_3_-CNCs as initial precursor from several TEM grids with morphological dimensions in similar range (scale bar: 1μm).*

***
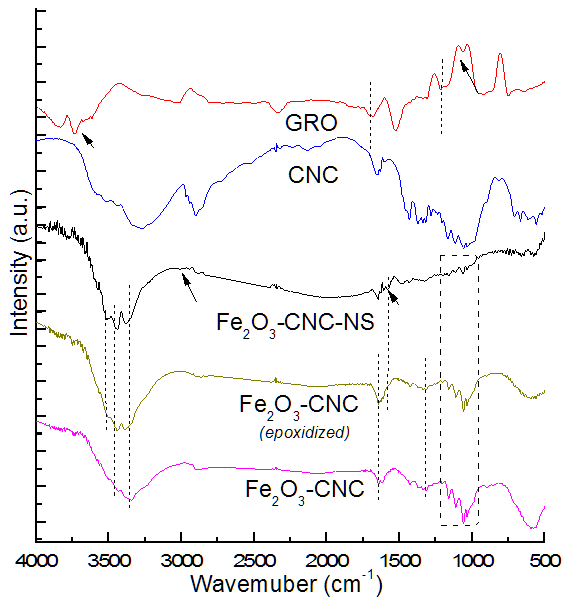

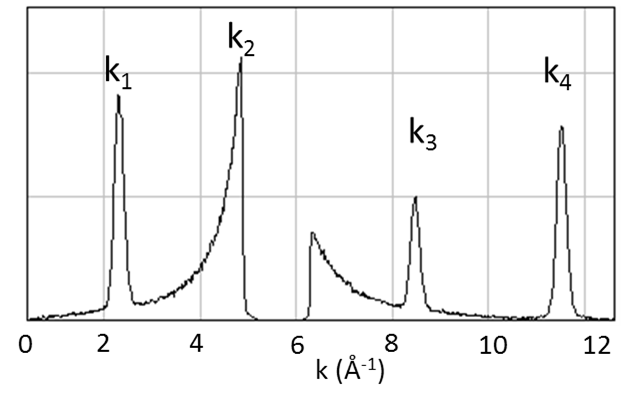
***

**(c)**

**(b')**

1

2

3

4


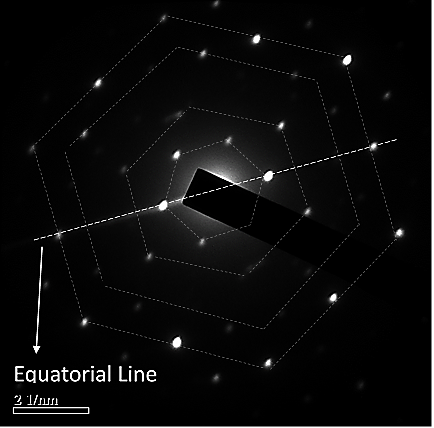

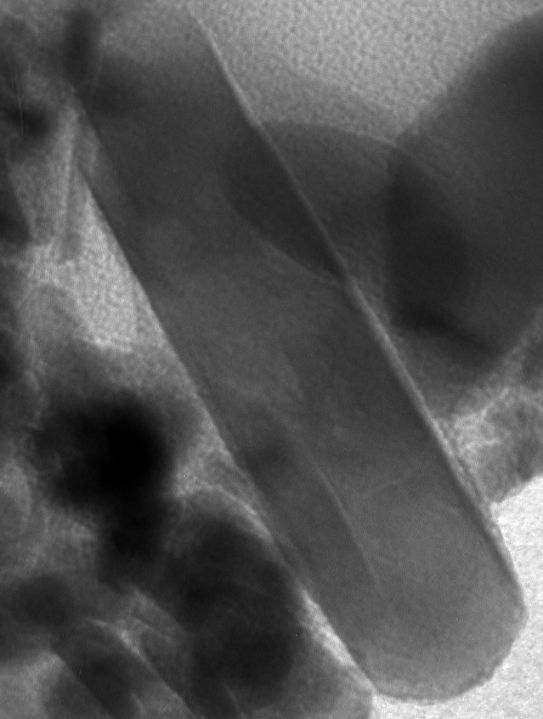


**(a)**

**(b)**

### *
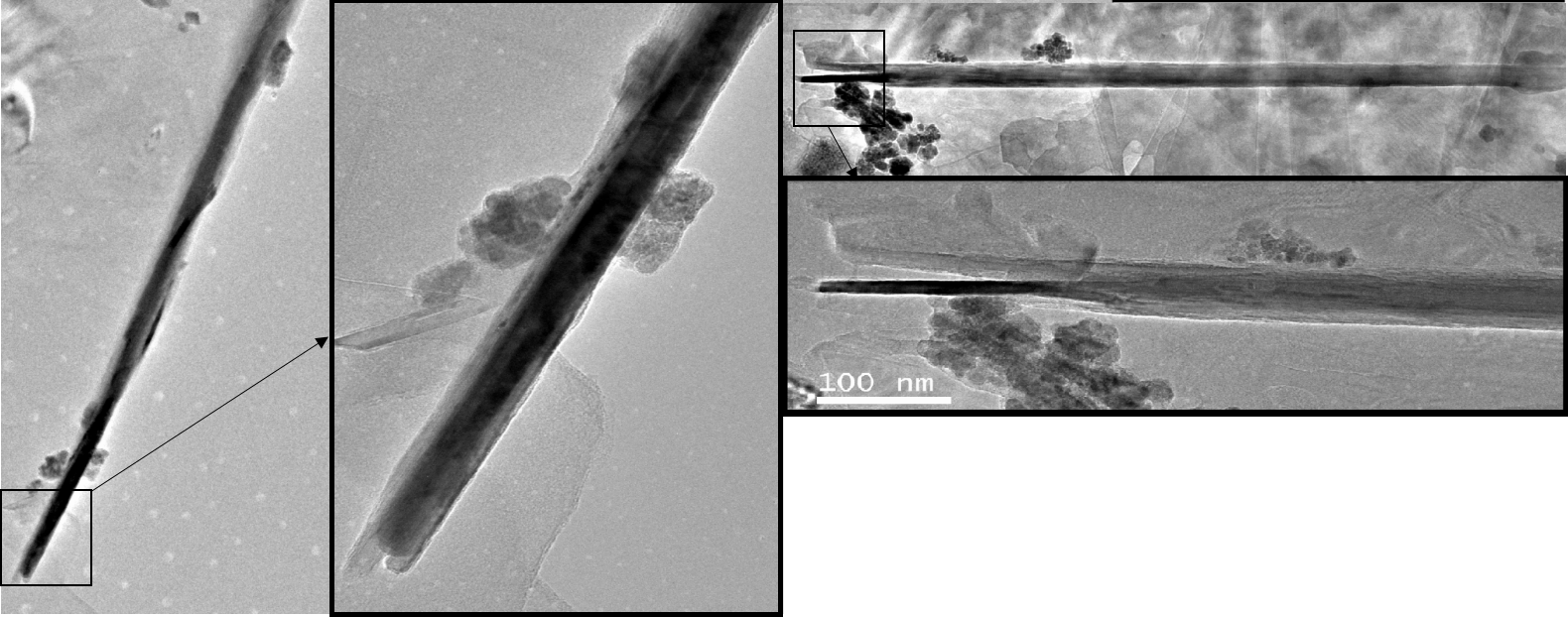
*


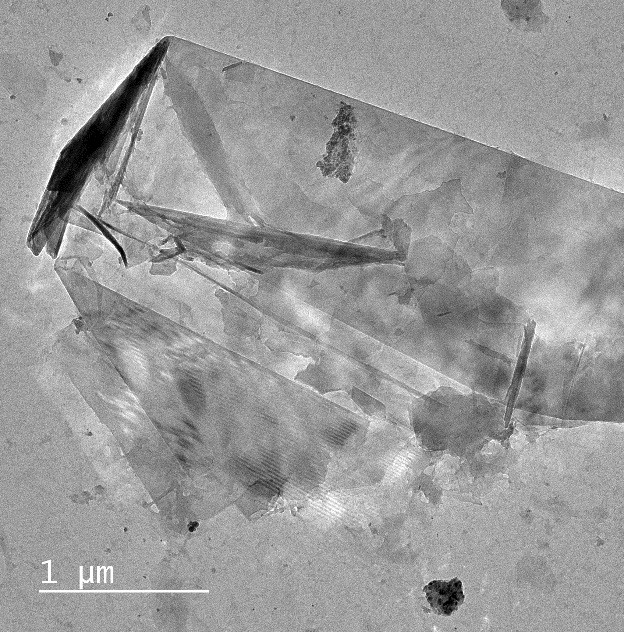

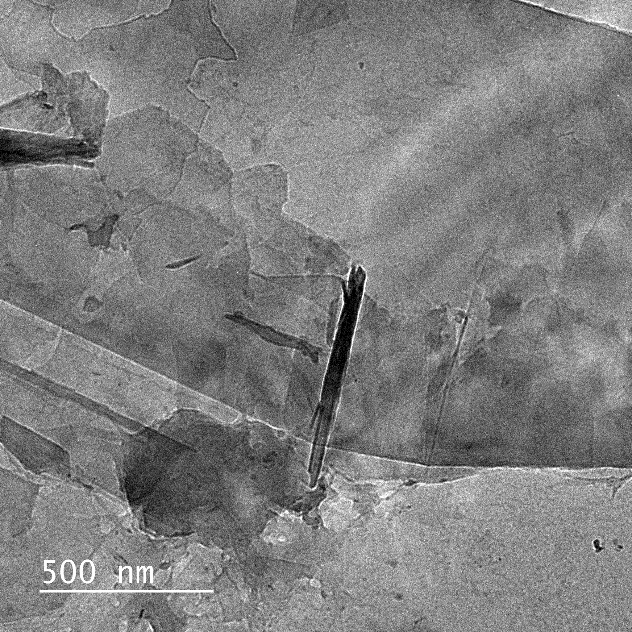


**Unfolded Graphene Sheets**

Bunch of CNCs lying on Graphene

Bunch of CNCs

**Absence of Scrolling**

**(d)**

**(d')**

**(e)**

**(f)**

### ***Figure S5:*** *(a) TEM micrograph, (b) selected area diffraction (SAED) pattern of Fe_2_O_3_-NS (designated area shown in black rectangle) and (b')* *Experimental equatorial line profile of intensities (measured along the blue-line marked in Figure S5 (b)). (c) Comparison of the FTIR spectra of CNCs, Fe_2_O_3_-CNC, epoxidized Fe_2_O_3_-CNC and Fe_2_O_3_-CNC-NS (GNS with Fe_2_O_3_-CNCs incorporated in it) to confirm the interfacial interaction of the graphene sheets with Fe_2_O_3_-CNCs resulting in the formation of GNS, (d) TEM micrographs showing the complete absence of any scrolling phenomenon in presence of the CNCs and (d')High resolution TEM micrographs shows the bunch of CNCs lying on the surface of graphene sheets, (e) and (f) TEM micrographs of Fe_2_O_3_-CNC-NS showing the presence of the CNCs (marked with black arrows) in the GNS.*

### ***Part II:*** *Fabrication and characterizations of Fe_2_O_3_-CNCs chemically modified with Pd and Pt nanoparticles (Pt/Pd-Fe_2_O_3_–CNCs).*

The spherical Pd and Pt nanoparticles are precipitated onto Fe_2_O_3_-CNCs in presence of reducing agents, which led to its uniform dispersion on its surface. From Fig. S6(a) and (c), Pd- Fe_2_O_3_-CNC and Pt-Fe_2_O_3_-CNCs show distinct fiber-like morphology with the Pd and Pt nanoparticles adsorbed on Fe_2_O_3_-CNCs surface as shown by the dark circles. High resolution FESEM micrographs (with magnification >125,000 X, Fig. S6(b)) shows the presence of the distinct spherical nanoparticles (bright spots marked with dotted circles) which are in size range of 25- 30nm due to the presence of agglomerates. Due to introduction of lower loading of the palladium and platinum salts, the adsorbed spherical Pd and Pt nanoparticles are uniformly distributed on the surface of Fe_2_O_3_-CNCs in form of small patches. Both the Pd- Fe_2_O_3_-CNC and Pt-Fe_2_O_3_-CNCs didn’t show any significant variation in terms of morphological dimensions and distribution of the adsorbed metallic nanoparticles. Due to high contrast of metallic Pt/Pd nanoparticles, they are clearly visible in TEM micrographs (shown in Fig. S6(f) and(g)) as dark dots in comparison to the CNC substrate due to its low contrast are not clearly visible in the background ^4^. However, the presence of rod-like alignment for both Pt/Pd and Fe_2_O_3_ nanoparticles confirms the presence of CNCs as substrate otherwise the nanoparticles would have been randomly distributed in the micrographs. TEM micrographs also reveals the similar fiber-like morphology of CNC substrate on which the spherical Pd and Pt nanoparticles are adsorbed on its surface with morphological dimensions in size range of 4-9nm and 6-11nm, respectively (shown in Fig. S6 (f') and (g')).

Fig. S6 (d), shows the XRD diffractogram of the CNCs with well resolved peaks at 2Ɵ=14.7°, 16.5°, 22.6° and 34.0° which corresponds to the crystallographic planes (101), (101̅), (002) and (040) respectively. The diffraction patterns for the fabricated nanocomposites of Pd- Fe_2_O_3_-CNC and Pt-Fe_2_O_3_-CNCs shows presence of peaks corresponding to CNCs, Fe_2_O_3_ nanoparticles alongwith the Pd and Pt nanoparticles distinctively. Both Pd- Fe_2_O_3_-CNC and Pt- Fe_2_O_3_-CNCs show the presence of peaks corresponding to cellulose I crystal structure which remains unaltered after the precipitation reactions. The diffractogram of Pd- Fe_2_O_3_-CNC, shows the presence of peaks at 2Ɵ=14.7°, 16.5°, 22.6° and 34.0° corresponding to the CNC crystal planes (101), (101̅), (002) and (040) respectively, peaks at 2Ɵ=39.9°, 46.4° and 68.3° represents the (111), (200) and (220) crystal planes of Pd nanoparticles respectively and with low intensity peaks at 2Ɵ=35.3° and 43.5° suggests the presence of the Fe_2_O_3_  nanoparticles with (311) and (400) crystallographic planes respectively. Similarly for the case of Pt-Fe_2_O_3_-CNCs, XRD diffractograms shows presence of peaks at 2Ɵ=14.9°, 16.6°, 22.7° and 34.2° representing the CNC crystal planes (101), (101̅), (002) and (040) respectively, peaks at 2Ɵ=39.8°, 46.5° and 68.2° signifies the (111), (200) and (220) crystal planes of Pt nanoparticles respectively, alongwith, the presence of low intense peaks at 2Ɵ=35.1° and 43.8° corresponding to (311) and (400) crystallographic planes of the Fe_2_O_3_  nanoparticles. The minimum crystallite size of the Pd and Pt nanoparticles adsorbed onto the surface of CNCs (calculated by Scherrer’s equation) are found to be ~5.35nm and 7.35nm respectively (corresponding to the peak at 2Ɵ=39.8° (111)), which is in line with the measured nanoparticle dimensions from the corresponding TEM micrographs. From the XRD diffractograms, it is interesting to observe that the peaks corresponding to Fe_2_O_3_ crystal planes underwent substantial reduction in its intensity but for CNCs it almost remains unaltered. This suggests that precipitation of the Pd/Pt nanoparticles probably took place onto the Fe_2_O_3_ nanoparticles present in Fe_2_O_3_-CNCs rather than the backbone of the CNCs which remains intact. This is probably due to the absence of hydroxyl groups of CNCs in the Fe_2_O_3_-CNCs which is already occupied by the adsorbed Fe_2_O_3_ nanoparticles at very high loading fractions (~35 wt. %). Due to absence of hydroxyl functionality, the precipitation of Pd/Pt salts are governed by the Fe_2_O_3_ nanoparticles which provides the sites for adsorption of metal salts followed by its reduction which is a electrochemically favourable process due to lower standard reduction potential ^56^. To further confirm the reduction of Pt and Pd nanoparticles onto the surface of Fe_2_O_3_-CNCs in case of Pd-Fe_2_O_3_-CNCs and Pt-Fe_2_O_3_-CNCs, it is studied through the crystal planes observed in high resolution-TEM (HR-TEM) micrographs. The HR-TEM images for the Pd-Fe_2_O_3_-CNCs shows the presence of crystal lattice fringes with interlayer spacing of ~0.200nm and ~0.222nm corresponding to (111) and (200) planes of Pd nanoparticles especially present on the outer layers (as marked in Figure S5(h) and(i)). The inner dark regions shows the presence of lattice plane (311) for Fe_2_O_3_ nanoparticles with inter-layer spacing of ~0.250nm as also evident from the XRD diffractograms which shows a small intensity peak at~35.2°(311). Similarly for the case of Pt-Fe_2_O_3_-CNCs, the (111) crystal planes of Pt nanoparticles are observed at the edges and (311) crystal lattice of Fe_2_O_3_ nanoparticles at the inner regions with an interlayer spacing of 0.230nm and 0.250nm respectively. For both the Pd/Pt-Fe_2_O_3_-CNCs, HRTEM micrographs shows the presence of crossover of lattice fringes which suggest that Pt/Pd nanoparticles have successfully adhered to the surface of Fe_2_O_3_ nanoparticles. Therefore, it is expected that during the chemical reduction of the PdCl_2_/PtCl_2_ salts, the Pt/Pd nanoparticles coalesces onto the surface of the Fe_2_O_3_ nanoparticles due to improved electrochemical interactions. Similar observation of fabricating nickel nanoparticles onto the surface of ZVI nanoparticles was studied by Li et al. ^6^ in which ZVIs acted as an adsorbent as well as reductant simultaneously. Therefore, from the crystallographic studies it can be concluded that both Pd/Pt nanoparticles can be supported onto the surface of Fe_2_O_3_-CNCs with the uniform size distribution which amalgamates to form a bimetallic structure.

Fig. S6 (e), shows the VSM plots for magneto-responsive Fe_2_O_3_-CNCs modified with the catalytically active Pd and Pt nanoparticles. Due to incorporation of the non-magnetic Pt and Pd species the magnetization values decreased to ~26.4 and 19.4 emu/g for the Pd-Fe_2_O_3_-CNCs and Pt-Fe_2_O_3_-CNCs respectively. The slight variation in the magnetization values is probably due to the difference in size of Pt and Pd nanoparticles adsorbed onto CNCs. Both Pd-Fe_2_O_3_-CNCs and Pt- Fe_2_O_3_- CNCs shows the presence of ferromagnetic behaviour as prevalent in the initial Fe_2_O_3_- CNCs as precursor. However, magnetization values obtained with Pd-Fe_2_O_3_-CNCs and Pt- Fe_2_O_3_- CNCs, alongwith, their distinct fiber/rod like-morphology provides enough momentum to the graphene sheets such that it could roll up their surface to form graphene nanoscrolls with encapsulated metallic nanoparticles, as discussed in subsequent sections.


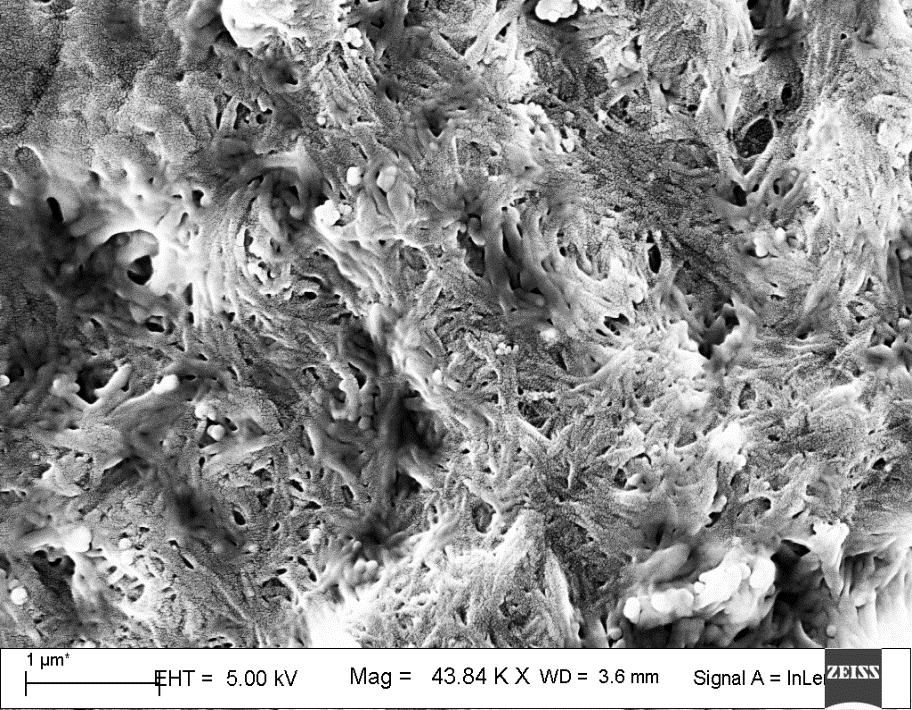


**(a)**


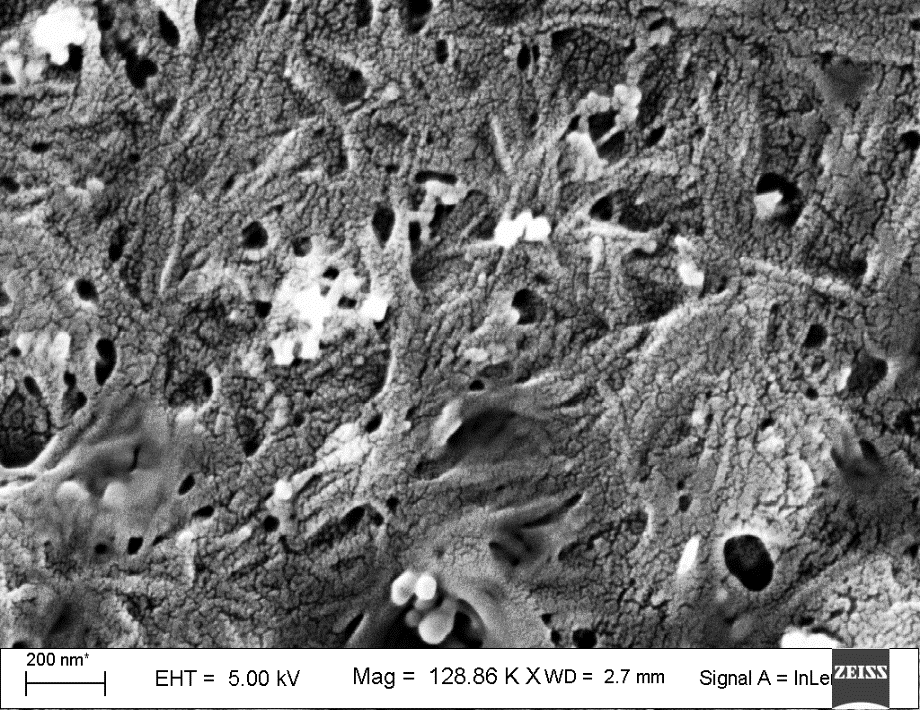


**(b)**


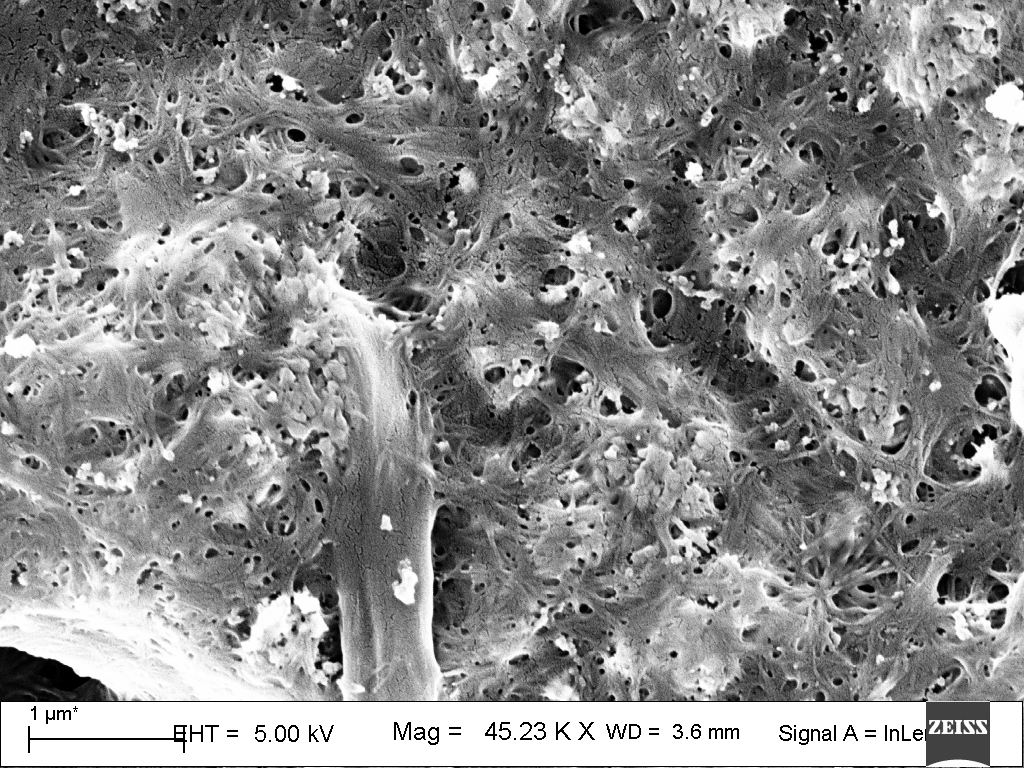


**(c)**


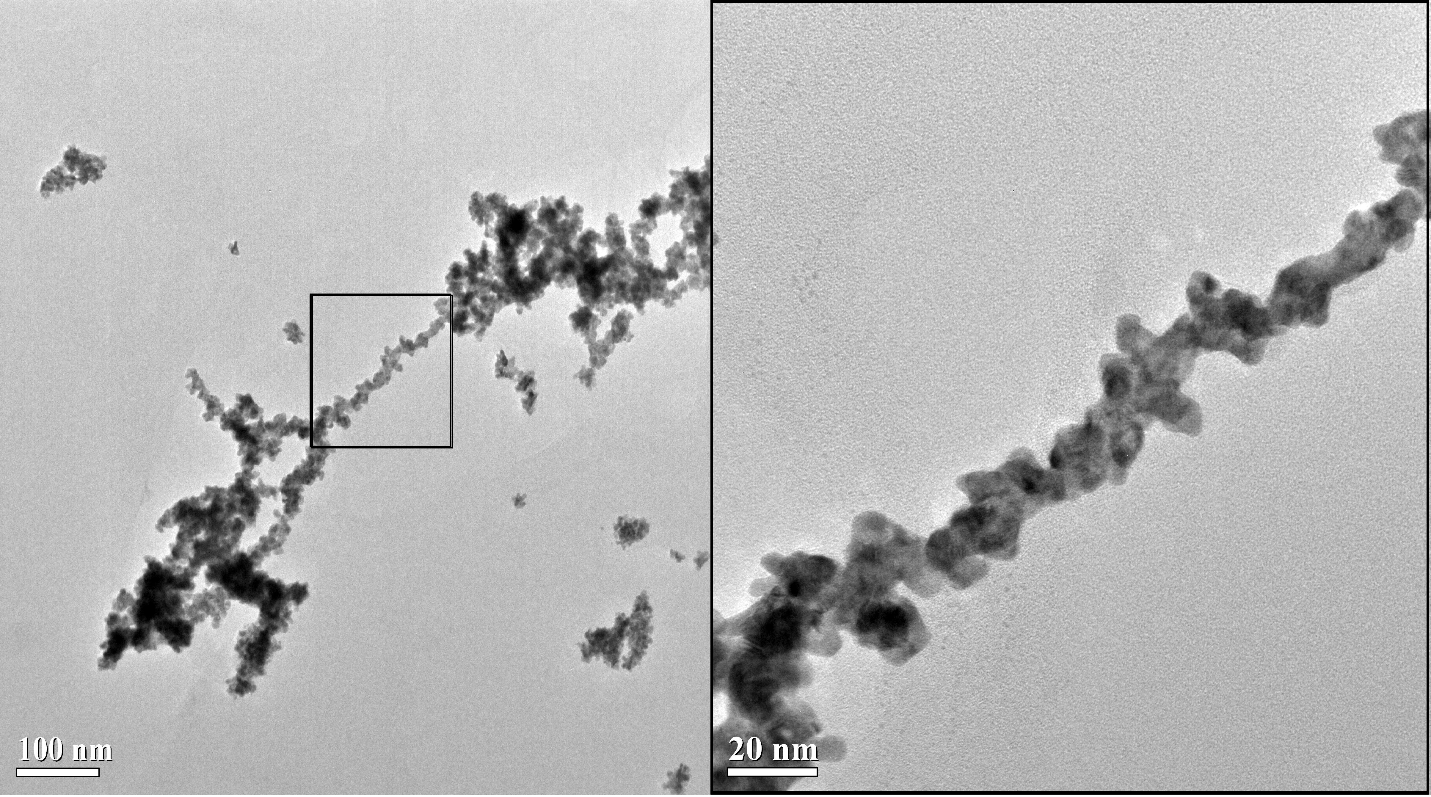


**(f)**

**(f')**


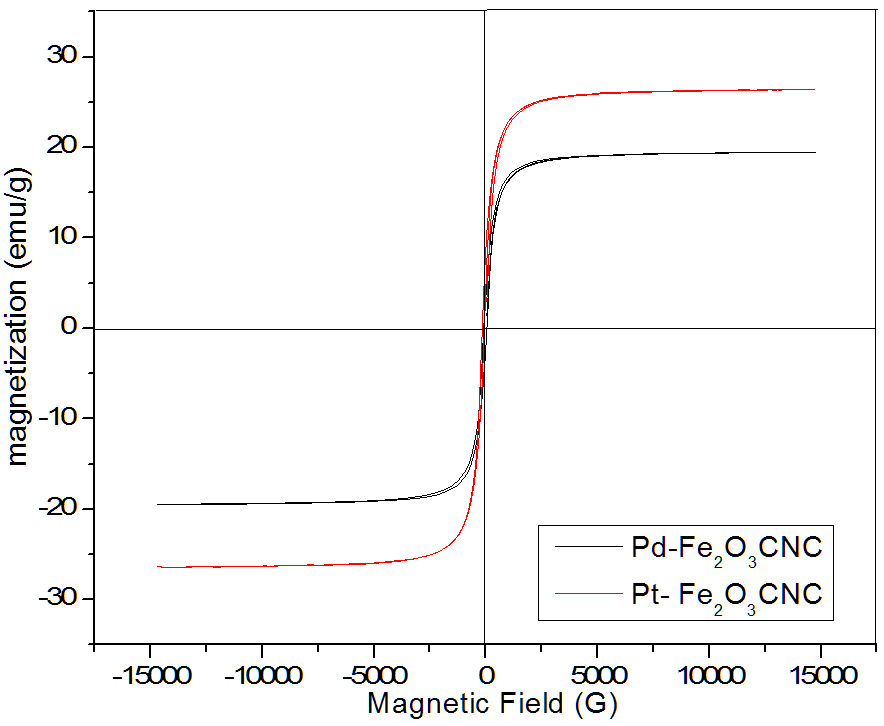


**(e)**


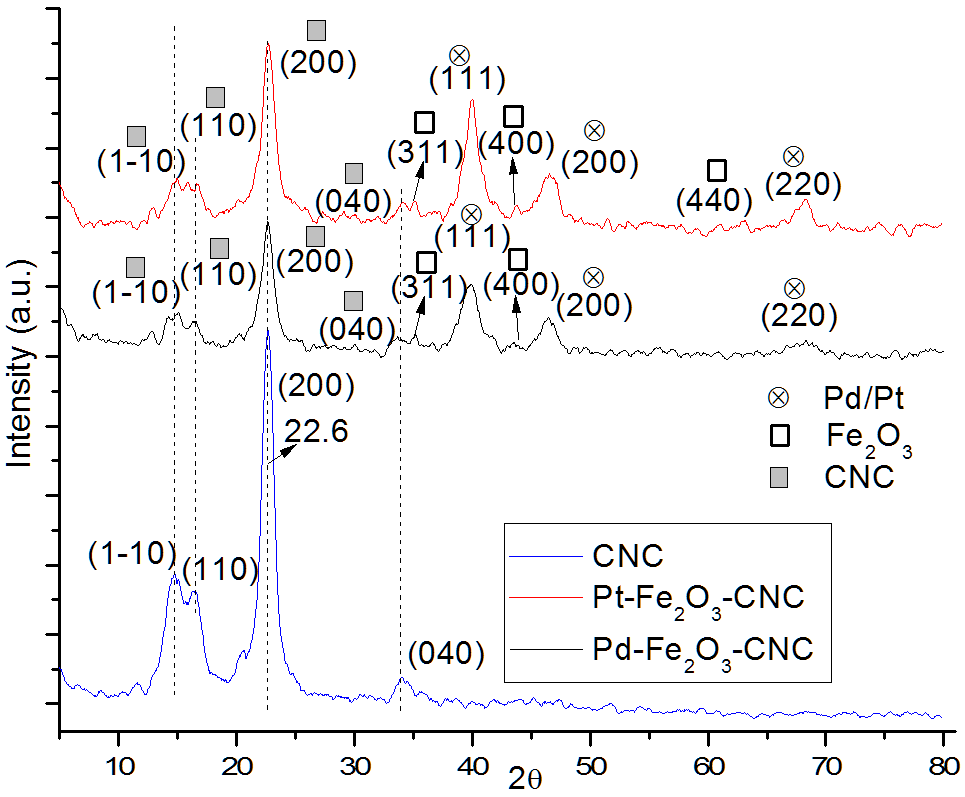


**(d)**


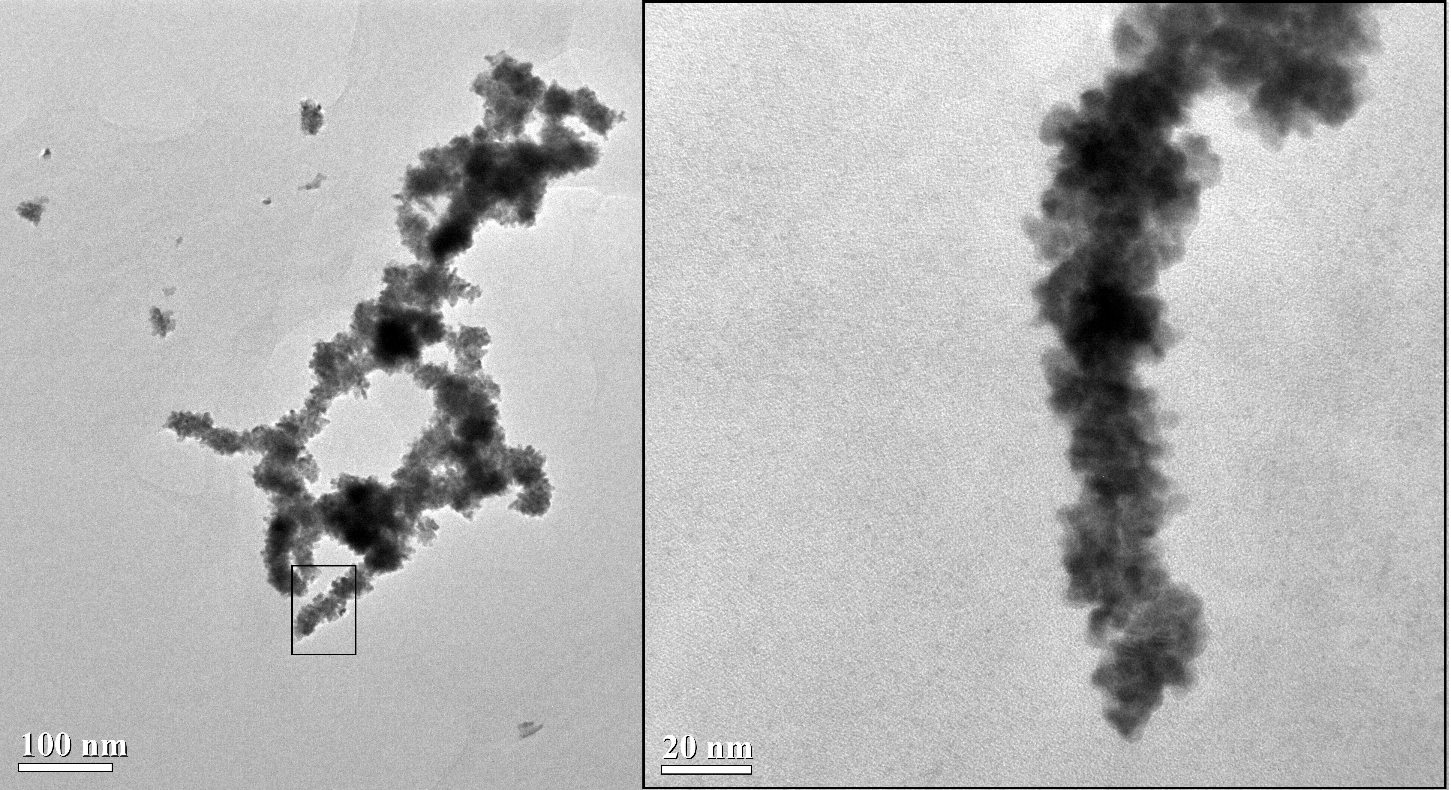


**(g)**

**(g')**


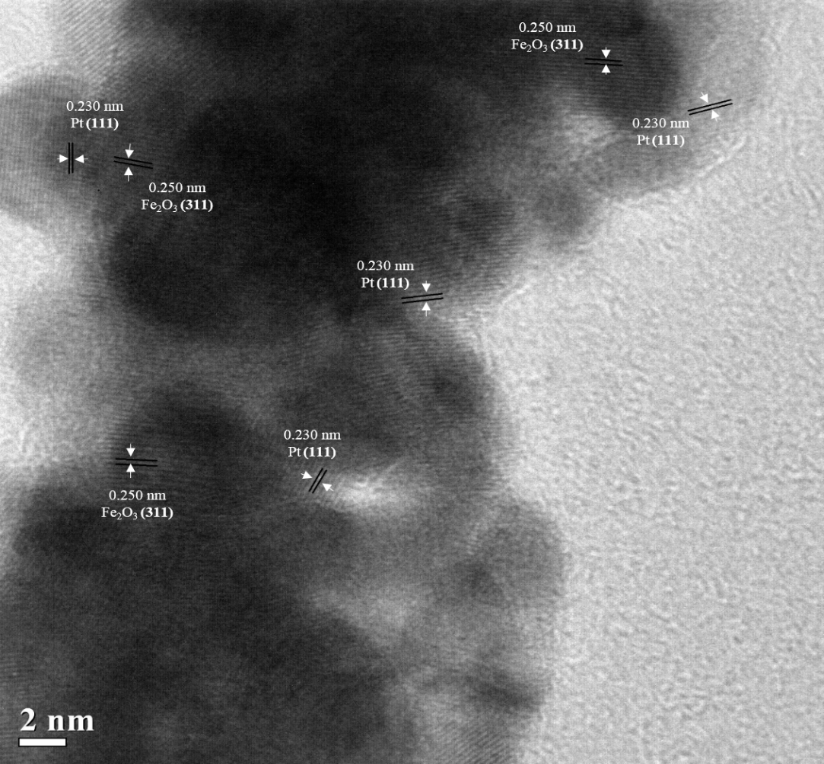


**(h)**


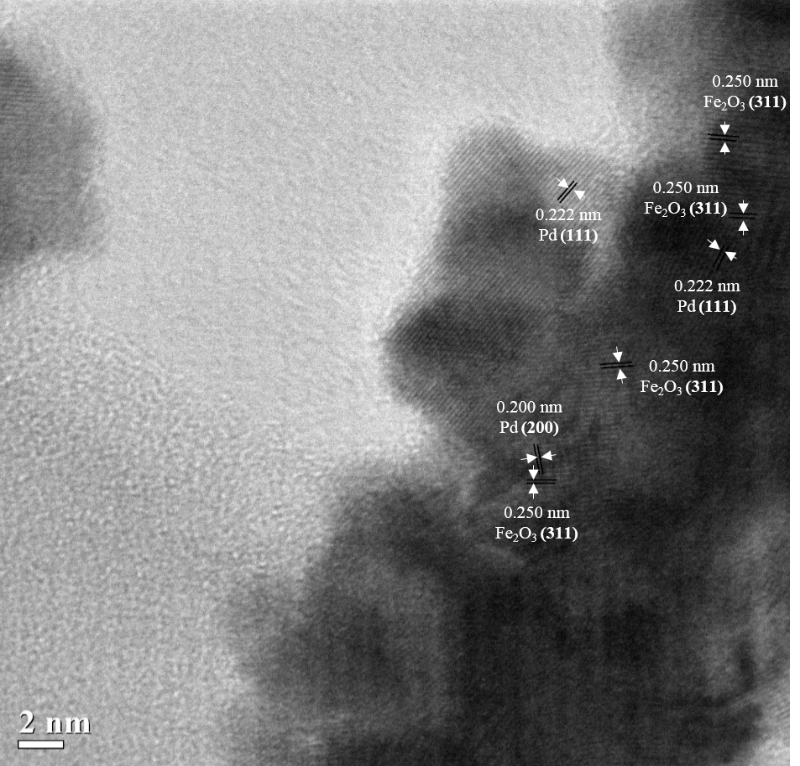


**(i)**

***Figure S6:*** *FESEM micrographs of the fabricated (a) Pd-Fe_2_O_3_-CNC with (b) high resolution micrographs of Pd-Fe_2_O_3_-CNC (at a resolution of ~125KX) which shows the presence of fine spherical Pd nanoparticles adsorbed onto Fe_2_O_3_-CNCs, (c) FESEM micrographs of Pt-Fe_2_O_3_-CNC, (d) XRD patterns of Pd-Fe_2_O_3_-CNC, Pt-Fe_2_O_3_-CNC and CNC, (e) Magnetic hysteresis loop for the fabricated Pd-Fe_2_O_3_-CNC and Pt-Fe_2_O_3_-CNC measured at 298K. TEM micrographs of (f) Pt-Fe_2_O_3_-CNC and (g) Pd-Fe_2_O_3_-CNC with the inset (f') and (g') showing the high resolution micrographs for the selected region in rectangular block which shows the presence of spherical Pd and Pt nanoparticles adsorbed onto CNCs. HRTEM micrographs of (h) Pt-Fe_2_O_3_-CNC and (i) Pd-Fe_2_O_3_-CNC with the respective crystal planes and lattice spacings of Pd and Fe_2_O_3_ planes marked in white.*


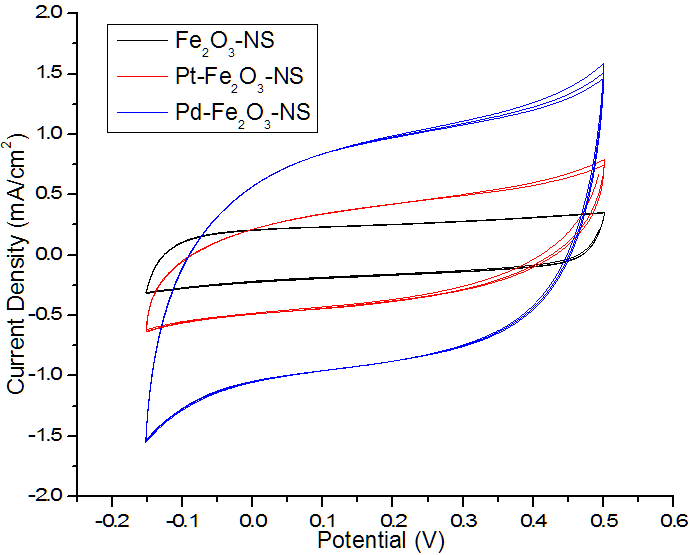


**(a)**


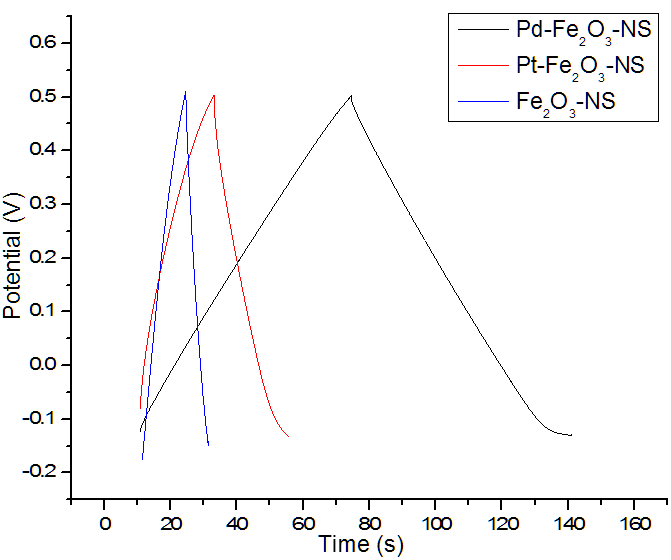


**(b)**

**(b)**

***Figure S7****: Comparisons of the (a) Cyclic Voltammograms of Pt-Fe_2_O_3_-NS, Pd-Fe_2_O_3_-NS and Fe_2_O_3_-NS measured at a scan rate of 100 mV/s after 10000 cycles, (b) charge-discharge curves of Pt-Fe_2_O_3_-NS and Pd-Fe_2_O_3_-NS measured at a current density of 1mA/cm^2^ and (c) cyclic stability tests of Fe_2_O_3_-NS measured at current density of 1A/g.*


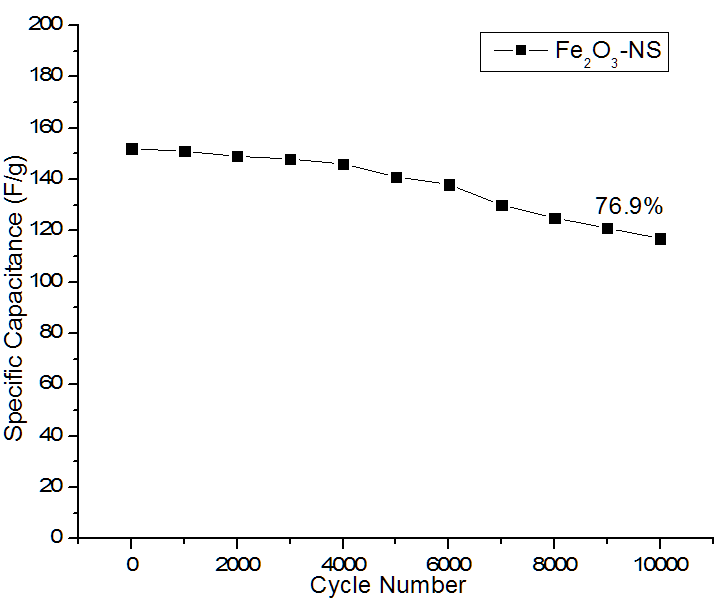


**(c)**

**References**

1. Zhang, X., Niu, Y., Meng, X., Li, Y. & Zhao, J. Structural evolution and characteristics of the phase transformations between α-Fe_2_O_3_ , Fe_3_O_4_ and γ-Fe_2_O_3_ nanoparticles under reducing and oxidizing atmospheres. *CrystEngComm* **15,** 8166–8172 (2013).

2. Dhar, P., Kumar, A. & Katiyar, V. Magnetic Cellulose Nanocrystals-based Anisotropic Polylactic Acid Nanocomposite Films: Influence on Electrical, Magnetic, Thermal and Mechanical Properties. *ACS Appl. Mater. Interfaces* (2016). doi:10.1021/acsami.6b02828

3. Dhar, P., Bhasney, S. M., Kumar, A. & Katiyar, V. Acid functionalized cellulose nanocrystals and its effect on mechanical, thermal, crystallization and surfaces properties of poly (lactic acid) bionanocomposites films: A comprehensive study. *Polymer* **101,** 75–92 (2016).

4. Rezayat, M., Blundell, R. K., Camp, J. E., Walsh, D. A. & Thielemans, W. Green One-Step Synthesis of Catalytically Active Palladium Nanoparticles Supported on Cellulose Nanocrystals. *ACS Sustain. Chem. Eng.* **2,** 1241–1250 (2014).

5. Huang, K.-C. & Ehrman, S. H. Synthesis of Iron Nanoparticles via Chemical Reduction with Palladium Ion Seeds. *Langmuir* **23,** 1419–1426 (2007).

6. Li, X. & Zhang, W. Iron Nanoparticles:  the Core−Shell Structure and Unique Properties for Ni(II) Sequestration. *Langmuir* **22,** 4638–4642 (2006).
